# Interactions between carnivore species: limited spatiotemporal partitioning between apex predator and smaller carnivores in a Mediterranean protected area

**DOI:** 10.1186/s12983-023-00489-w

**Published:** 2023-05-25

**Authors:** Francesco Ferretti, Raquel Oliveira, Mariana Rossa, Irene Belardi, Giada Pacini, Sara Mugnai, Niccolò Fattorini, Lorenzo Lazzeri

**Affiliations:** 1 Research Unit of Behavioural Ecology, Ethology and Wildlife Management, Department of Life Sciences, University of Siena, Via P.A. Mattioli 4, 53100 Siena, Italy; 2 NBFC, National Biodiversity Future Center, 90133 Palermo, Italy; 3 CESAM, Department of Biology, University of Aveiro, Campus de Santiago, 3810-193 Aveiro, Portugal

**Keywords:** Interspecific coexistence, Interspecific interactions, Temporal partitioning, Carnivores, Competition

## Abstract

**Background:**

There is need of information on ecological interactions that keystone species such as apex predators establish in ecosystems recently recolonised. Interactions among carnivore species have the potential to influence community-level processes, with consequences for ecosystem dynamics. Although avoidance of apex predators by smaller carnivores has been reported, there is increasing evidence that the potential for competitive-to-facilitative interactions is context-dependent. In a protected area recently recolonised by the wolf *Canis lupus* and hosting abundant wild prey (3 ungulate species, 20–30 individuals/km^2^, together), we used 5-year food habit analyses and 3-year camera trapping to (*i*) investigate the role of mesocarnivores (4 species) in the wolf diet; (*ii*) test for temporal, spatial, and fine-scale spatiotemporal association between mesocarnivores and the wolf.

**Results:**

Wolf diet was dominated by large herbivores (86% occurrences, *N* = 2201 scats), with mesocarnivores occurring in 2% scats. We collected 12,808 carnivore detections over > 19,000 camera trapping days. We found substantial (i.e., generally ≥ 0.75, 0–1 scale) temporal overlap between mesocarnivores—in particular red fox—and the wolf, with no support for negative temporal or spatial associations between mesocarnivore and wolf detection rates. All the species were nocturnal/crepuscular and results suggested a minor role of human activity in modifying interspecific spatiotemporal partitioning.

**Conclusions:**

Results suggest that the local great availability of large prey to wolves limited negative interactions towards smaller carnivores, thus reducing the potential for spatiotemporal avoidance. Our study emphasises that avoidance patterns leading to substantial spatiotemporal partitioning are not ubiquitous in carnivore guilds.

**Supplementary Information:**

The online version contains supplementary material available at 10.1186/s12983-023-00489-w.

## Background

Apex predators include keystone species that play fundamental roles in ecosystems through direct and/or indirect effects on organisms belonging to lower trophic levels [78]. Although the study of interspecific interactions of large carnivores has typically involved predator–prey relationships, there is an increasing interest on the interactions that apex predators establish with smaller carnivores [19, 27, 70, 75]. These interactions can influence population dynamics of smaller carnivores, with potential effects across trophic levels [19, 70, 78]. This is especially relevant in the face of the increasing threats to the persistence of most large carnivores [78] or, conversely, considering the recent recovery of apex predators in temperate countries [15]. Apex predators can affect mesocarnivores through lethal interactions involving intraguild predation or non-consumptive killing [27, 70]. The fear of larger predators can influence behaviour and ecology of subordinate carnivores, which are expected to avoid larger ones, leading to spatial and/or temporal partitioning (e.g., [28, 43, 71, 96]). Smaller carnivores may also be facilitated through increased foraging opportunities determined by carcasses of apex predators’ prey [1, 84, 88]. Both suppressive and facilitative interactions may impact species at lower trophic levels, triggering a top-down force shaping carnivore communities [27, 75].

Describing the intensity and outcome of spatiotemporal interactions among carnivore species can be far from trivial [82]. The effects of apex predators over smaller carnivores can switch from positive (i.e., facilitation) to negative (i.e., competition or predation) ones often on a site-specific basis related to factors such as predator density and resource availability [5, 39, 42, 75]. Since competition is emphasised by resource scarcity [7], the potential for negative vs. positive/neutral interactions should rise with decreasing availability of prey to dominant predators, in turn stimulating the propensity of apex predators to consider smaller carnivores as potential competitors [70]. Abundance of large herbivores is also expected to influence the attraction of apex predators to alternative food resources, which would influence their tendency to consider smaller carnivores as suitable prey. If so, the way smaller carnivores perceive larger ones as potential dangers would not be consistent across spatial and temporal scales, influencing the potential for interspecific spatial/temporal avoidance vs. overlap.

Integrating multiple dimensions of the ecological niche is needed to combine data on spatial and temporal interactions, as well as the contribution of mesocarnivores to apex predator diet. Camera trapping studies are typically conducted over short term temporal scales that are especially suitable for species inventories or monitoring purposes, but may not disclose interspecific interactions over long periods of time. Thus, studies integrating information on apex predator diet and its spatiotemporal interactions with smaller species over long study periods are still scarce. We contribute to filling this gap by using intensive food habit (5 years) and camera trapping (3 years) data to investigate interactions between an apex predator (the grey wolf *Canis lupus*) and mesocarnivores (the red fox *Vulpes vulpes*, the European badger *Meles meles*, the stone marten *Martes foina* and the pine marten *Martes martes*). We hypothesise that, in an area where large prey is available at high densities, mesocarnivores play a negligible role in the wolf diet (e.g., [21, 55, 59]), and no major temporal and spatial partitioning occurs between the apex predator and smaller carnivores.

We worked in a Mediterranean protected area recently recolonised by the wolf [34, 36, 80]. Three species of wild ungulates at high densities live in the area, i.e., the wild boar *Sus scrofa*, the fallow deer *Dama dama* and the roe deer *Capreolus capreolus* (c. 20–30 individuals/100 ha, together, [34]. A preliminary, two-year study of wolf food habits showed that wild ungulates were the staple prey of wolves (*c.* 80–85% of occurrences, volume, or biomass in diet), with mesocarnivores occurring in *c.* 4% wolf scats [34]. Among them, the badger was reported 3.3% times in wolf diet, much more than foxes and *Martes* spp. (0.3% times, each of them [34]). Previous work also suggested a substantial temporal overlap between the wolf and the red fox [36, 80]. The latter showed greater use of large ungulate carcasses with respect to times when the wolf did not occur in the area, suggesting a potential trophic facilitation [36].

We attempted to assess interspecific interactions on multiple levels [32]. We have assessed (*i*) the contribution of mesocarnivores to the diet of the apex predator, and (*ii*) temporal, spatial, and fine-scale spatiotemporal relationships between the apex predator and mesocarnivores. Predation would be expected to stimulate fear reactions in the preyed species. If so, we would expect a stronger avoidance of wolves by badgers than by the other two mesocarnivores. Based on the literature and preliminary information on our study system, as well as local high density of large prey [34], we predicted (*1*) a scarce occurrence (i.e., lower than 5%, overall) of mesocarnivores in the wolf diet, with a relative greater occurrence of the badger rather than the fox and stone/pine marten [34]. With respect to wolf-fox interactions, we predicted (*2*) a significant association between their temporal activity patterns and spatial variation of detection rates [36, 80]. If so, we expected that interspecific temporal overlap (*2a*) was high (i.e., greater than 0.75 on a 0–1 scale, sensu [62] and (*2b*) greater in sites with high wolf activity than in sites less used by wolves, that (*2c*) spatial variation of fox detection rates was associated with that of wolf, and (*2d*) no support for interspecific segregation at a finer spatiotemporal scale. With respect to wolf-badger interactions, we expected a major potential for interspecific avoidance [25]. If so, we predicted (*3a*) a moderate temporal interspecific overlap (i.e., included between 0.50 and 0.75, [62], being (*3b*) greater in sites with low wolf activity than in high wolf sites, (*3c*) with no support for either spatial association of detection rates or (*3d*) finer-scale spatiotemporal association. With respect to wolf-*Martes* spp., we did not expect a substantial spatiotemporal partitioning. Thus, we predicted (*4a*) a high temporal overlap (i.e., greater than 0.75, [62], (*4b*) consistent between sites with high and low wolf activity, with (*4c*) no negative relationship between the spatial variation of their detection rates and (*4d*) no support to finer-scale spatiotemporal segregation.

## Results

### Mesocarnivores in wolf diet

**Fig. 1:**
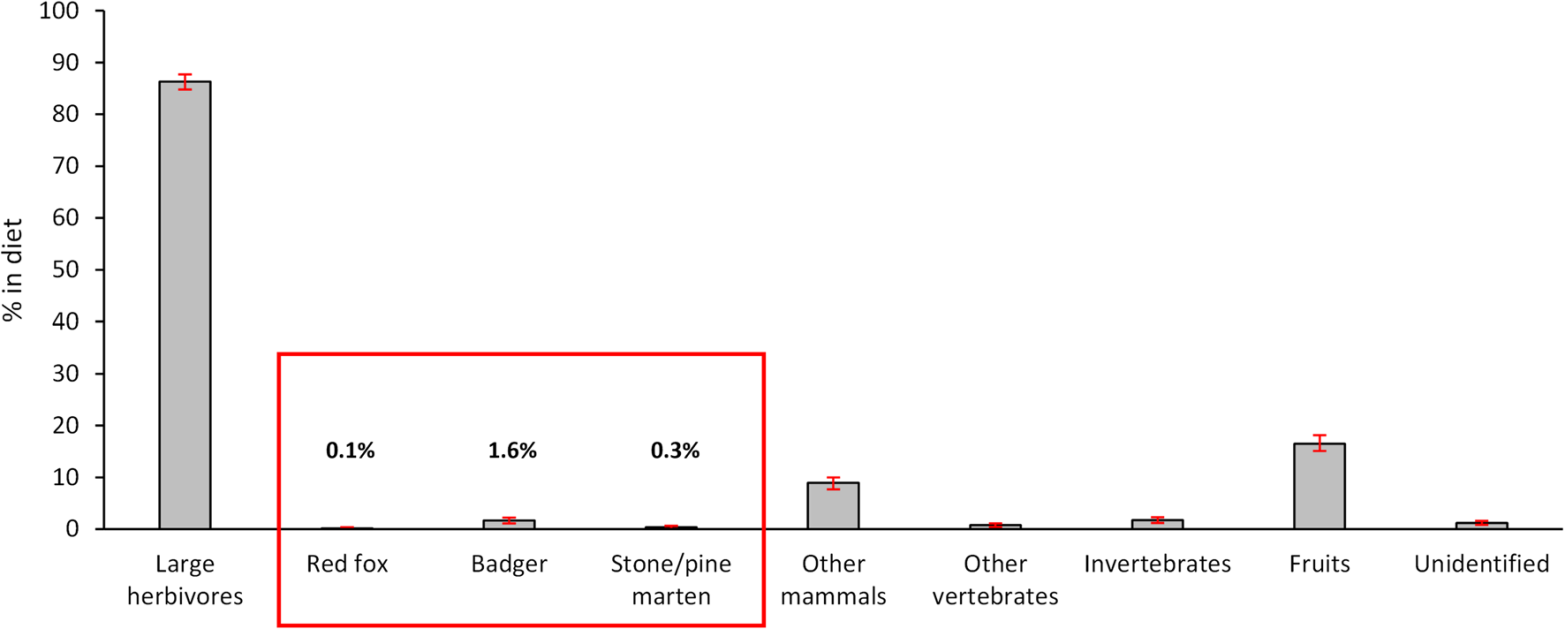
Food habits of the wolf (April 2016–March 2021, *n* = 2201 scats): percentage in diet of different food categories expressed as absolute frequency of occurrence. Error bars indicate 0.95 bootstrapped confidence intervals estimated through 1000 replicates. In the red box, our focal mesocarnivore species. Large herbivores include wild boar, fallow deer, roe deer and livestock (i.e., cattle and sheep). Other mammals include coypu, crested porcupine, European brown hare, domestic cats, and smaller rodents

Wolf diet was dominated by large herbivores (86.3% occurrence; Fig. 1). Red fox and *Martes* remains were detected in just three and seven out of 2201 scats, respectively (0.1% and 0.3% occurrence, respectively; Fig. 1). The badger was reported with an overall frequency of 1.6% (6.9% in the first year, then 1.2–1.7% yearly). Other mammals and fruits occurred in 8.9% and 16.5% scats, respectively.

### Temporal interactions

**Fig. 2:**
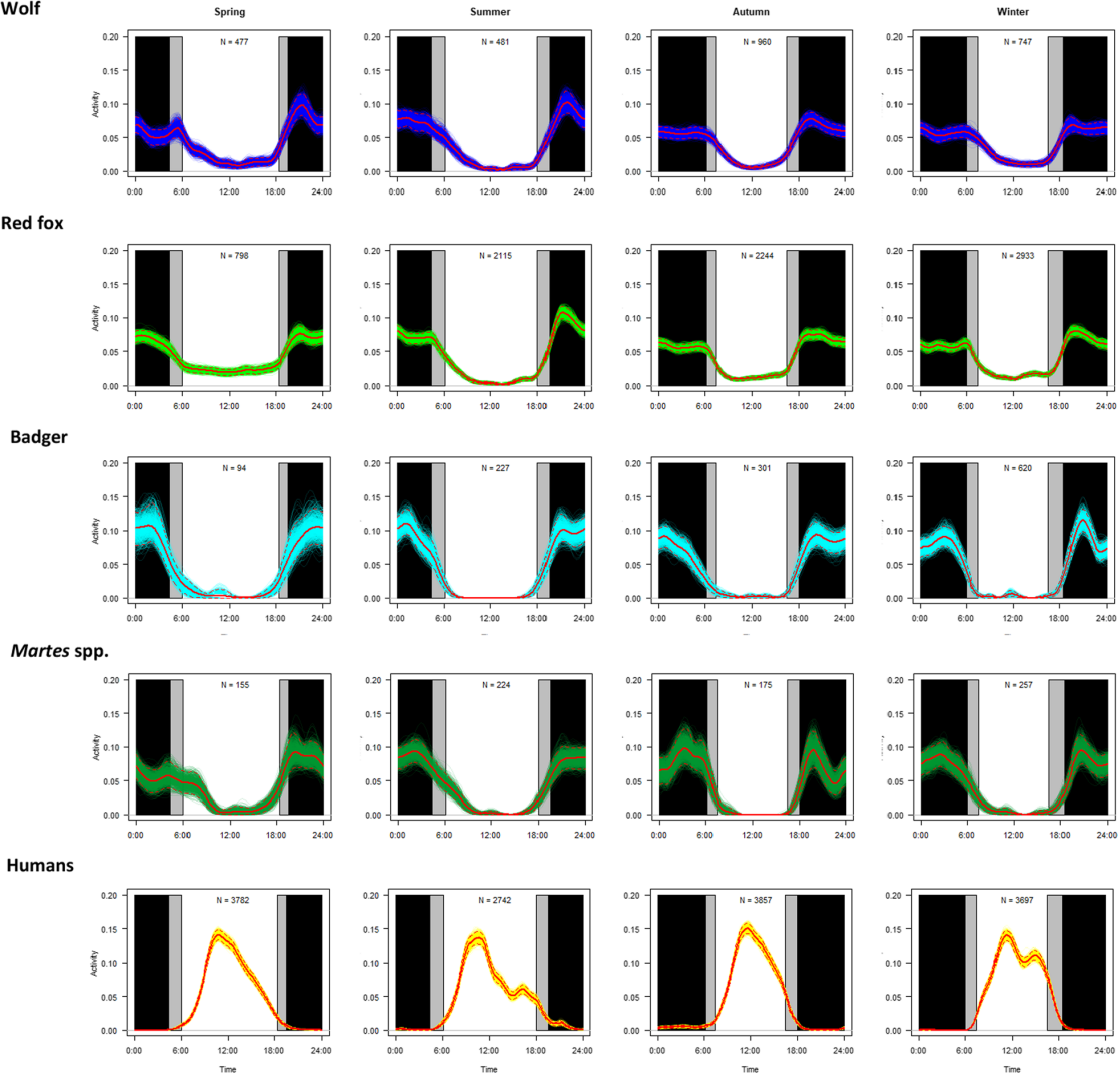
Temporal activity patterns of the wolf, red fox, badger, stone/pine marten and humans at seasonal scale (spring: April–June; summer: July–September; autumn: October–December; winter: January–March) in 2017–2021. Red solid lines indicate estimates of temporal activity patterns through kernel density estimators: the area under the function obtained corresponds to the probability of observing the animal for each period. Colored lines represent bootstrapped estimates of activity patterns through 1000 replicates; dashed red lines represent 0.95 bootstsrapped confidence intervals. Grey rectangles indicate times of day of dawn and dusk during each season; black rectangles indicate times of day preceding the dawn and following the dusk. Sample size (i.e., number of detections) is shown in each panel

All the carnivore species showed nocturnal activity patterns, whereas human activity was concentrated during daylight (Fig. 2; see Additional file 1 for analyses on separate years). There was only slight support for a difference in temporal activity patterns of the red fox and those of the wolf, for which a significant difference was found only in two seasons out of twelve (spring of first and third year: *U* = 0.218–0.285, *p* < 0.05; Additional file 2). There was strong support for a difference between temporal activity patterns of the badger and those of the wolf in the second and third year, as well as in the spring of the first year, with the former being strictly nocturnal and the latter showing some levels of activity during the day/dawn/dusk (*U* = 0.229–1.422, *p* < 0.05; Additional file 2). There was slight support for a difference in temporal activity patterns of *Martes* spp. from those of wolves, with significant differences reported in five out of twelve seasons (*U* = 0.195–0.537, *p* < 0.05; Additional file 2). Each year and for each species, there was strong support for a difference between carnivore temporal activity patterns and those of people (*U* = 0.408–76.832, *p* < 0.001).

**Fig. 3:**
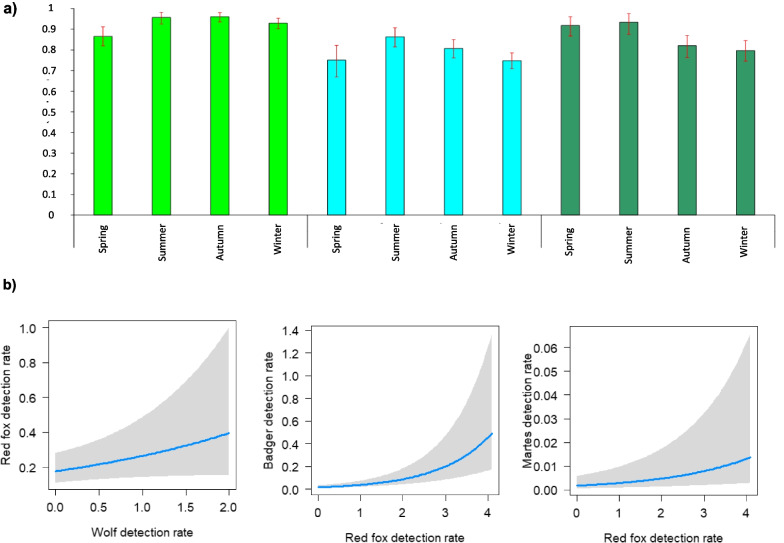
**a** Temporal overlap between the wolf and mesocarnivores estimated through nonparametric coefficient of overlap (∆4), at the seasonal scale (2017–2021). Error bars indicate bootstrapped 0.95 confidence intervals obtained through 1000 replicates. Dark blue: wolf; light green: red fox; light blue: badger; dark green: stone/pine marten. **b** Monthly spatial variation of detection rates (i.e., number of detections per site per day) of mesocarnivores, in 2017–2021): red fox in relation to wolf and badger detection rates; badger in relation to red fox detection rate; stone/pine marten in relation to red fox detection rate. Blue lines indicated fitted relationships estimated through generalised linear mixed models with negative binomial errors. Grey areas indicate 0.95 confidence intervals of fitted relationships

Interspecific temporal overlap between the wolf and mesocarnivores showed ∆_4_ coefficients ≥ 0.75 (wolf-fox: 0.87–0.96; wolf-badger: 0.75–0.86; wolf-*Martes* spp.: 0.80–0.93) (Fig. 3a). Overlap with people was low (∆_4_ = 0.04–0.27). These findings were confirmed by analyses conducted in separate years (see Additional file 4). Coefficients of overlap between the wolf and mesocarnivores were generally higher than or close to 0.75 in both “high wolf” and “low wolf” sites (Additional file 4). Differences between sites were consistently lower than 5% with largely overlapping 0.95 confidence intervals, except for the fox and the badger in spring and winter, and for the *Martes* spp. in autumn: in these cases, coefficients of overlap with the wolf were *c.* 7–43% higher in “high wolf” than in “low wolf” sites (Additional file 4).

### Spatial interactions

There was only weak support for a positive relationship between fox and wolf detection rates: confidence intervals of the estimated coefficient were close to ‘0’ in the best and in the second selected models (Table 2; Additional file 7; Fig. 3b). Fox detection rate was the lowest in spring and increased with badger detection rates (Table 2). Fox detection rates were the lowest in the third study year, but this effect was not supported by the analysis of a subset of data collected in the central-northern sector of the study area sampled since the first study year (see “Temporal and spatial interactions: data collection” section) (Table 2; Additional file 7).

For the European badger, the selected model included the positive effects of red fox detection rates and shrub cover (Tables 1, 2; Fig. 3b). The best model also included the negative effect of human detection rates, although confidence intervals of the estimated coefficient were very close to ‘0’, suggesting weak support (Tables 1, 2). There was support to higher detection rate in winter than in the other seasons (Table 2).

**Table 1:** Model selection for spatial variation of carnivore detection rates

Response variable	Model	Variables	*K*	logLik	AICc	ΔAICc	Weight
Red fox detection rate	Best	Wolf + Badger + Season + Study year	**11**	**− 2456.999**	**4936.3**	**0.00**	**0.453**
	Second	Wolf + Badger + Season	**9**	**− 2459.525**	**4937.3**	**0.95**	**0.282**
	Third	Badger + Season + Study year	**10**	**− 2458.863**	**4938.0**	**1.68**	**0.197**
	Fourth	Badger + Season	8	**− **2461.959	4940.1	3.78	0.068
Badger detection rate	Best	Red fox + Humans + Season + Shrub cover	**10**	**− 1095.246**	**2210.8**	**0.00**	**0.813**
	Second	Red fox + Season + Shrub cover	9	**− **1098.073	2214.4	3.60	0.135
	Third	Red fox + Humans + Season	9	**− **1099.134	2216.5	5.73	0.045
	Fourth	Red fox + Season	8	**− **1102.213	2220.6	9.84	0.007
*Martes* spp. detection rate	Best	Red fox + Humans + Study year + Season + Canopy cover	**12**	**− 823.646**	**1671.7**	**0.00**	**0.514**
	Second	Red fox + Study year + Canopy cover + Season	**11**	**− 825.350**	**1673.0**	**1.35**	**0.261**
	Third	Red fox + Humans + Study year + Season	11	**− **826.199	1674.7	3.05	0.112
	Fourth	Red fox + Humans + Canopy cover + Season	10	**− **827.237	1674.7	3.07	0.112

Summary of model selection. Variables influencing spatial variation of detection rates of red fox, Eurasian badger and *Martes* spp., estimated through Generalised Linear Mixed Models. Predictors included in selected models are shown. All models included the random effects of camera trapping location and camera model; the log(number of sampling days) was included as offset variable. For the red fox, three models were selected; for the badger, only the best model was selected; for *Martes* spp. the first two models were selected. All selected models were shown in bold. The second, third and fourth ranked models were also shown for comparison purposes. Parameters of models selected after the first are shown in the Additional file 6

**Table 2:** Factors influencing spatial variation of carnivore detection rates

Analysis	Species	Variable	*B*	S.E	0.95 CIs	
					**−**	+
Spatial interactions	Red fox	Intercept	**− **0.994	0.252	**− **1.488	**− **0.500
		Wolf	0.082	0.043	**− **0.002	0.167
		Badger	0.239	0.047	0.146	0.332
		Study year [Second]	**− **0.251	0.152	**− **0.548	0.046
		Study year [Third]	**− **0.342	0.154	**− **0.644	**− **0.040
		Season [Summer]	0.438	0.108	0.226	0.650
		Season [Autumn]	0.235	0.109	0.022	0.448
		Season [Winter]	0.384	0.111	0.167	0.600
	Badger	Intercept	**− **3.611	0.184	**− **3.971	**− **3.250
		Red fox	0.424	0.057	0.312	0.536
		Humans	**− **0.240	0.107	**− **0.450	**− **0.030
		Season [Summer]	0.115	0.182	**− **0.241	0.471
		Season [Autumn]	0.034	0.177	**− **0.313	0.380
		Season [Winter]	0.552	0.174	0.212	0.892
		Shrub cover	0.347	0.120	0.112	0.583
	*Martes* spp.	Intercept	**− **2.937	0.431	**− **3.782	**− **2.092
		Red fox	0.261	0.068	0.129	0.394
		Humans	**− **0.132	0.104	**− **0.336	0.071
		Season [Summer]	**− **0.175	0.193	**− **0.554	0.204
		Season [Autumn]	**− **0.615	0.197	**− **1.000	**− **0.229
		Season [Winter]	**− **0.399	0.195	**− **0.781	**− **0.016
		Study year [Second]	**− **0.766	0.288	**− **1.331	**− **0.201
		Study year [Third]	**− **0.458	0.299	**− **1.043	0.128
		Canopy cover	0.371	0.165	0.049	0.694
Spatiotemporal interactions	Wolf- Red fox	Intercept	1.915	0.086	1.746	2.083
		Wolf-Fox vs. Fox-Wolf	**− **0.053	0.079	**− **0.209	0.102
	Wolf- Badger	Intercept	2.179	0.187	1.813	2.546
		Wolf-Badger vs. Badger-Wolf	0.092	0.205	**− **0.310	0.494
	Wolf- *Martes* spp.	Intercept	2.249	0.756	0.766	3.731
		Wolf-*Martes *vs.* Martes*-Wolf	**− **0.102	0.487	**− **1.057	0.853

Variables influencing spatial variation of detection rates of red fox, badger and *Martes* spp., as well as spatiotemporal patterns of each mesocarnivore species in relation to the wolf. Spatial variation of detection rates was estimated through Generalised Linear Mixed Models with negative binomial errors. Variables included in the best models are shown. Estimated coefficients and their standard error as well as 0.95 confidence intervals are shown. Spatiotemporal patterns were estimated through Linear mixed models with gaussian errors

For *Martes* spp., the best model included the effects of red fox detection rate, human detection rate, canopy cover, season as well as study year; the same variables were included in the second best model, with the exception of human detection rate (Table 1; Additional file 6). *Martes* spp. detection rate increased with canopy cover; a positive association was supported also with red fox detection rate, whereas confidence intervals for human detection rates included ‘0’ (Table 2; Fig. 3b; Additional file 6). A greater detection rate was found in spring–summer than in the other seasons, as well as in the first year than in the second-third years, and this effect was confirmed also after subsetting the dataset including only observations collected in the same sector over three years (Additional file 7).

### Spatiotemporal interactions

After applying our filtering criteria, these analyses were based on 1602 temporal distances between wolves and red foxes, 198 temporal distances between wolves and badgers, as well as 56 temporal distances between wolves and *Martes* spp. Spatiotemporal analyses did not provide support for differences between temporal distances of mesocarnivore detections after wolf detections and temporal distances of wolf detections after mesocarnivore ones (Table 2).

## Discussion

Avoidance of larger predators has been suggested to play a major role in shaping spatial and/or temporal habits of mesocarnivores, to limit the risk of encounters with larger competitors/predators, thus potential killers [27, 70, 75]. Our findings support only minor spatiotemporal partitioning between mesocarnivores and an apex predator. Wild ungulates were abundant in our study area, with overall summer densities ranging between 22 and 31 individuals per km^2^, which presumably led wolves to concentrate on these larger and remunerative prey (see also [67]. Conversely, smaller carnivores provided only a scarce contribution to the diet of the wolf, except for the badger. A potential caveat to our conclusions is that wolves may play a greater contribution to mesocarnivore mortality than it could be estimated through dietary analyses. In fact, interspecific killing can be the outcome of competitive—and not consumptive—processes [70]. Nevertheless, at least for foxes a potential for dietary facilitation has been detected in our study area, with a substantial interspecific spatiotemporal overlap with the wolf, no evidence for avoidance and support to temporal synchronisation [36, 80]. Data based on GPS telemetry would help assessing the contribution of wolves to mesocarnivore mortality, as well as spatiotemporal partitioning acting at finer spatial and/or temporal scales.

All carnivores showed nocturnal or crepuscular habits and therefore a substantially high interspecific temporal overlap between mesocarnivores and the wolf was detected. These results may suggest a common avoidance of daylight as a strategy to limit encounters with humans [37, 66, 92]. In turn, human activity may reduce options for carnivores to increase their diurnal activity to limit predation/interference risk [61, 63]. However, nocturnal activity of carnivores was generally consistent across sites in our study area. Although detection rates at camera trapping sites may underestimate the effects of human disturbance, human activity was rather concentrated in a small number of locations. The three sites most used by people totalised *c.* 40% human detections, with *c.* 75% detections being concentrated in just 10 camera trapping locations, resulting in vast sectors of our study area relatively less disturbed. Moreover, human activity is especially concentrated in spring–summer, i.e., the touristic season, and it consists especially of recreational activity with weak impact on wildlife and in particular hiking, biking, and running. We found consistent interspecific temporal overlap between the wolf and mesocarnivores between sites characterised by high vs. low human activity. Furthermore, the results agree with temporal activity patterns reported in the literature (e.g., wolf: [18, 91], red fox: [14, 24], badger: [45, 95], *Martes* spp.: [64, 94]). Nocturnal activity patterns may reflect a common response of our study species to long-term coexistence with humans in an anthropised landscape. Nevertheless, our results did not support human recreational activities influencing the current spatial variation of temporal overlap between the wolf and mesocarnivores. We worked in a protected area where human activity is expectedly reduced in respect to unprotected sites, which may have led to a limited impact on interactions between mammalian species (but see [83]). A comparison of our results with study areas where human activity is not allowed, as well as with sites located outside protected areas, would help clarifying it.

Although the wolf could be a fox killer [70, 99], our 3-year dataset based on intensive camera-trapping indicates no evidence for either a temporal or a fine-to-coarse scale spatial avoidance of the former by the latter. Results agree with previous findings suggesting substantial temporal and spatial overlap between these two carnivores ([63, 95], see also [36, 80], for our study area). The red fox is a generalist carnivore showing a remarkably adaptable diet, ranging from invertebrates to small vertebrates and even ungulate offspring [49, 76]. In our study area, its trophic niche includes resources ranging from fruits to invertebrates, to small-medium-sized vertebrates and to ungulates [13, 36]. The red fox is a major consumer of carcasses of wolf prey [85, 101], potentially showing an overlap with the wolf diet concerning the use of ungulates [4, 73]. Preliminary work in our study area showed increased use of ungulate carcasses by foxes in respect to times when the wolf was absent [36]. Although fox density has been shown to be reduced by greater wolf pack size at local scales [99], a study conducted at a continental scale showed no negative effect of wolf density on fox abundance [72]. The size and direction of wolf-fox interactions are expected to be site-specific, depending on factors such as the availability of alternative resources, in turn affecting the potential for competitive interactions to occur [75]. We observed (*i*) a substantial interspecific temporal overlap, being also greater in sites with higher wolf detection rates than in “low wolf” sites [80], (*ii*) no support for negative spatial association of their detection rates, and (*iii*) no evidence for a fine-scale spatiotemporal avoidance. We also found no evidence for a decrease in fox detection rates throughout the study, which may be suggestive of no decrease in fox density along with the concurrent increase of wolf numbers (from one to two–three packs). Our results corroborate the hypothesis that foxes took benefit from wolf presence through increased availability of carcasses of wolf prey [36]. The potential for wolf-fox interactions—including also dietary relationships and overlap—to change along with temporal variations in wolf and prey numbers should be assessed.

We expected that the badger was the mesocarnivore species showing the greatest partitioning with the wolf, among our study species. Although this medium-sized mustelid showed the lowest temporal overlap with the wolf, no strong evidence supporting an avoidance of the apex predator was found. Similar findings were obtained for the *Martes* spp.. With respect to the badger, results were only partially consistent with findings from other areas of peninsular Italy, where a substantial temporal [95] or spatial [63] partitioning with the wolf was reported. Although our results may appear surprising, they suggest that partitioning with the apex predator was not based on strong spatiotemporal avoidance. The relatively greater occurrence of badgers than foxes and martens in the wolf diet might not necessarily be related to predation, but it could be the result of consumption of carcasses, e.g., at roadkills. However, badgers were detected by camera traps *c.* 6.5 times less often than foxes, which may suggest a much lower density which, in turn, would be unlikely to reflect a greater abundance of roadkills and carcasses of the former than the latter. We cannot rule out that coexistence may be favored by specific behaviours not detectable through our camera trapping study, i.e., fossoriality [25, 65] and semi-arboreal habits [6], for the badger and *Martes* spp., respectively. For example, it has been shown that badger setts in the areas more used by wolves were used *c*. 60% less often than those located in areas less used by wolves [25]. Our results showed that badger and *Martes* spp. detection rates increased with shrub cover and canopy cover, respectively. Shrub cover could be expected to provide concealment against predators. Forested habitats represent the optimum for *Martes* spp., which can find tree cavities required as reproduction and resting sites [6, 58].

Camera traps allow detecting animals during their movements on trails, which would underestimate the use of resting sites (e.g., dens, homesites, rendezvous sites) or trees (for *Martes* spp.). Moreover, considering the high local density of wolves (1–3 packs/year in our *c.* 90 km^2^ study area), the potential for mesocarnivores to spatially avoid wolves would be expected to be limited. In areas hosting high densities of large carnivores, mesocarnivores are expected to face chronical high risk of encounters with apex predators and may be forced to base avoidance tactics—if any—on immediate cues [26]. Moreover, risk-tolerant tactics would be favoured by the high availability of rewards, such as ungulate carcasses. Mechanisms of spatial avoidance acting at a finer scale may not be ruled out, and GPS telemetry [8, 20, 96] or experiments [26, 47, 100] are needed to test for them.

Apex predators can favour smaller carnivores by providing additional food resources through carcasses of their prey [1, 84, 88]. While the local importance of carcasses for the fox diet has been assessed [36], recent data are not available for badgers and martens. Locally, the badger has been reported to feed mainly on insects and fruits [16], and *Martes* spp. can show similar food habits [2, 54, 86]. Future work should test for the potential for carcasses of wolf prey to attract badgers and martens. The importance of subsidies by carrion is also related to the availability/quantity of alternative resources and may increase when other resources are scarce (‘*stress gradient hypothesis*’, [10, 89, 102]). Carnivores can reduce the risk of encountering larger competitors if they can exploit alternative resources, whereas the fear of starvation may overcome the fear of an apical predator [75]. This is in accordance also with the ‘*fatal attraction hypothesis*’ on carcasses, indeed the energetic rewards from scavenging in some cases may outweigh the risks of being killed [88]. Nevertheless, interactions among carnivores may show remarkably complex patterns, also including co-occurrence of significant lethal interference and attraction to carcasses, if the rewards of the latter outweigh the cost of the former [82].

Interactions among carnivores are complex and can switch from apparently positive to negative outcomes for the smaller species (e.g., [27, 70, 75, 82]). Our results emphasise that strong temporal and/or spatial partitioning may not be ubiquitous (see [20] for predator–prey relationships). We urge researchers to conduct longer-term, multi-dimensional studies [82] to disentangle mechanisms of coexistence that could act at finer-scale levels [26]. The ongoing expansion of the carnivore distribution range in western countries would offer such opportunity [15, 46].

## Conclusions

The potential for suppressive vs. facilitative interactions between carnivores has been shown to increase with latitude, likely determined by the decreasing environmental productivity—thus food availability—towards northern sites, in the boreal hemisphere [75]. In our Mediterranean study area, the availability of large, substantial prey to wolves was remarkably high, including three species of wild ungulates for an overall density of *c.* 20–30 individuals/km^2^ (see “Methods” section). The importance of preserving a rich and diverse prey community to enhance carnivore conservation has been largely emphasised, with prey depletion hampering carnivore persistence [12, 30], promoting predator interest to use livestock as an alternative prey [44, 60], and stimulating interspecific competition [35]. Access to a rich prey spectrum would make large carnivores not particularly interested to mesocarnivores as potential prey, as well as increasing their tolerance towards scavengers in proximity to carcasses. If so, a lack of strong avoidance patterns would depend mainly on a reduced fear of apex predators [26], although this hypothesis requires confirmation through experimental tests [26, 41, 100]. Potential changes of temporal and/or spatial association between carnivores along with variations in prey availability, wolf numbers, as well as gradients of human disturbance, should be assessed.

## Methods

### Study area

**Fig. 4:**
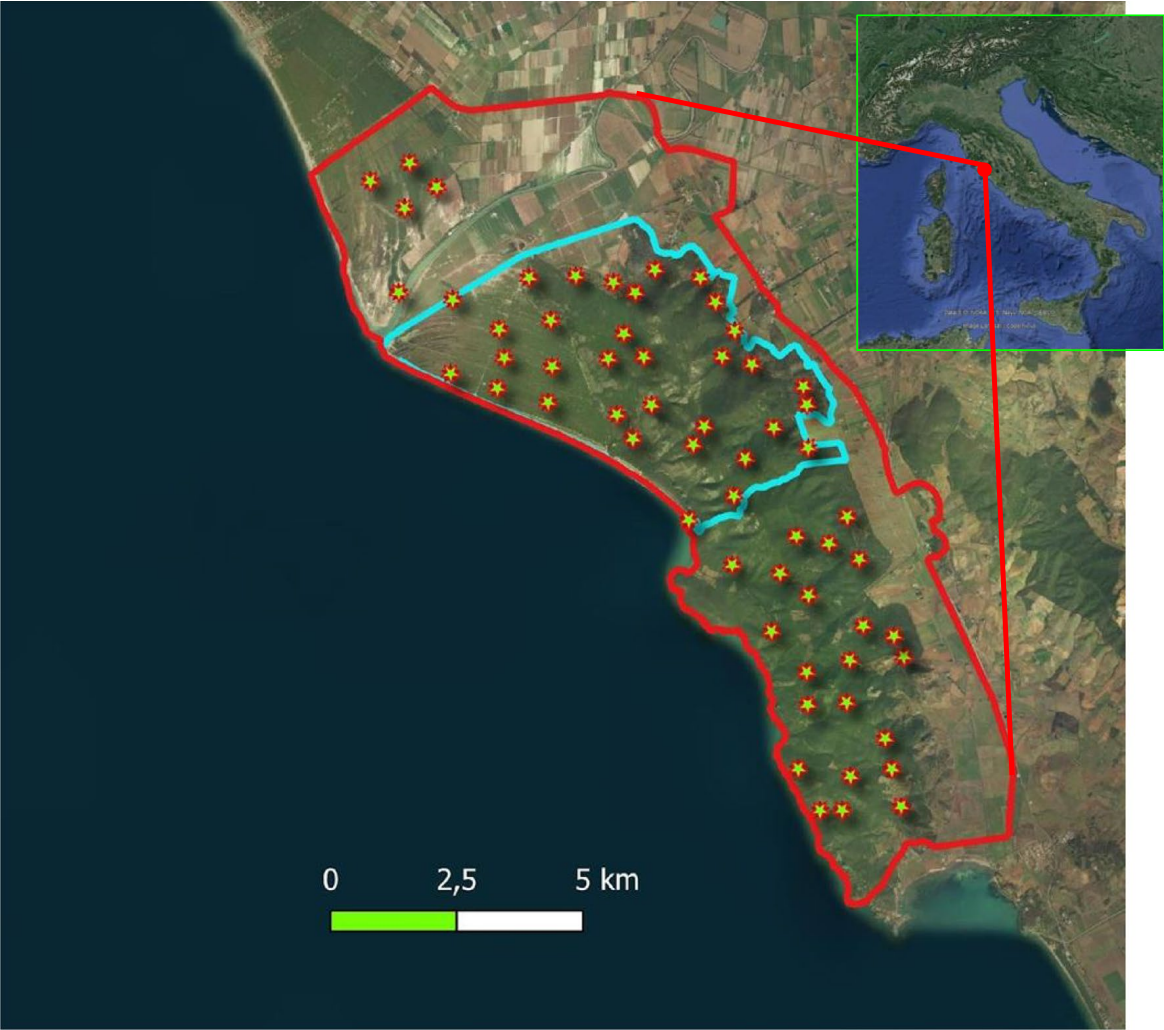
Map of the study area with locations of camera trapping sites (stars). The red line indicates the borders of Maremma Regional Park. The blue line indicates the borders of the study sector sampled during our first study year (October 2017–September 2018)

Our study was carried out in the Maremma Regional Park (central Italy, *c.* 90 km^2^; Fig. 4; 42.626371° N, 11.099303° E). The local climate is the Mediterranean, with hot-dry summers (mean daily temperature: 9–24 °C; monthly rainfall: 9.3 mm, in July, to 81.8 mm, in November, [36]. Vegetation is composed mainly of Mediterranean scrubwood (58%), including oakwood dominated by holm oak *Quercus ilex* trees, and shrubwood, with principally holm oak and strawberry tree *Arbutus unedo*, as well as bushes (holm oak, rosemary *Rosmarinus officinalis*, juniper *Juniperus spp.*, rockrose *Cistus* spp*.*). Other habitats are the pinewood (10%, mainly domestic pine *Pinus pinea*), abandoned olive groves and pastures (15%), set-aside grassland (4%), and crops (12%, mainly cereals and sunflower). For details on vegetation and habitats of our study area see Sforzi et al. [87] and Melini et al. [56].

A wolf pack was reported in the area in 2015, a second pack settled in 2017 [34], and a third pack has been reported since 2019–2020. Ungulates include the fallow deer, the wild boar, and the roe deer, for a total summer density ranging from *c.* 22 individuals/100 ha to *c.* 31 individuals/km^2^ (wild boar: 10.5–15.1 individuals/km^2^; fallow deer: 8.3–9.1 individuals/km^2^; roe deer: 3.1–6.9 individuals/km^2^; estimates obtained through feces counts: [31, 33, 34]). The area includes many medium-sized mammals, i.e., the crested porcupine *Hystrix cristata*, the coypu *Myocastor coypus*, the European brown hare *Lepus europaeus*, the red fox *Vulpes vulpes*, the European badger *Meles meles*, the wildcat *Felis silvestris*, the stone marten *Martes foina*, the pine marten *Martes martes*, and various species of smaller mammals. Livestock (*c.* 20 heads/km^2^) includes cattle and horses, roaming in sectors of pinewood and pastures, as well as two sheep herds in sectors of the agricultural area. Population control of wild boar and fallow deer is conducted under the responsibility of the Park Agency through culling (both species) and trapping (wild boar), to limit the negative impacts of these ungulates on habitats/species with conservation relevance, and on agriculture.

### Mesocarnivores in wolf diet

We assessed the frequency of occurrence of mesocarnivores in the wolf diet by analysing wolf food habits through the identification of indigested remains in scats ([17, 34, 36], for our study area). Wolf scats were collected monthly (April 2016–March 2021) along itineraries for a total of up to *c.* 120 km/month, and opportunistically during usual activities of territory patrolling by Park Wardens. Overall, 2201 scats were collected and used for analyses (April 2016–March 2017: *n* = 72; April 2017–March 2018: *n* = 347; April 2018–March 2019: *n* = 589; April 2019–March 2020: *n* = 594; April 2020–March 2021: *n* = 599). The content of each scat was assessed according to Lovari et al. [50, 51] through (*i*) a macroscopic comparison of hairs with a reference collection of hairs of potential wolf prey, using parameters such as color, shape, length, and thickness, and (*ii*) microscopic analyses of cuticle, medulla, and cortex, under an optical microscope (100–400×), through which hair features were compared with reference atlases, identification keys and reference collection of hair of local prey [22, 90]. For the purposes of this work, wolf prey was categorised as “large herbivores” (i.e., wild boar, fallow deer, roe deer, or livestock), “red fox”, “badger”, “*Martes* spp.”, “other mammals”, “other vertebrates”, “invertebrates”, “fruits” and “unidentified” items. For each *i-*th category, we calculated its frequency of occurrence in the diet as the percentage of scats including it. Then, we calculated bootstrap 0.95 confidence intervals of frequency of occurrence through 1000 replicates. For details, see Additional file 1.

### Temporal and spatial interactions: data collection

Spatiotemporal activity patterns of the wolf, red fox European badger, stone marten and pine marten were assessed through camera trapping, with locations defined within sampling grids (for our study area: [29, 36, 80]). Stone marten and pine marten were pooled together as *Martes* spp., owing to the potential problems in the correct identification of the two species [74, 79], especially for nocturnal videos. In a first study year (October 2017–September 2018), we concentrated on a *c.* 30 km^2^ study sector in the central-northern part of Uccellina hills and pinewood, used by the wolf pack that first settled in the area [36, 80]. A sampling grid (cell size: 1.3 km × 1.3 km) was superimposed on the study area through the QGIS software and 21 locations were defined according to Rossa et al. [80], with cameras being deployed along animal trails or forest roads. Cameras were rotated monthly across locations, to monitor all the locations for about 1 month/season (“autumn”: October–December,“winter”: January–March; “spring”: April–June; “summer”: July–September), i.e., each location was monitored c. 4 months throughout the study year (Fig. 4). In a second study year (April 2019–March 2020) we extended our study to cover the whole Uccellina hills, pinewood and Trappola area (*c*. 60 km^2^). We used a finer sampling grid with 1 km × 1 km cells and increased the number of locations to 57, with a monthly rotation of cameras. In a third study year (April 2020–March 2021) we added three more locations and kept all the 60 locations fixed all year long. Over our 3-year study period, we obtained 138 yearly deployments and 1032 monthly deployments (all years pooled).

Given constraints related to the nature of the terrain and the presence of dense scrubwood, we made an effort to put cameras at an average height of c. 30–100 cm, to increase the detection probability of smaller animals such as mustelids and foxes [97]. Thus, 88.4% of 138 yearly deployments occurred at a height lower than 100 cm from the ground. Considering the above-mentioned constraints, as well as to reach a compromise between visibility and reduction of risk of thefts, 10.1% of deployments had to occur at a height of 101–150 cm and 1.5% of deployments (*n* = 2) at a height greater than 150 cm. To account for these differences, we included camera height as predictor in statistical models (see below). Camera traps (models: Owlzer Guard Z2, Comitel Guard, Comitel Guard Micro 2, Ir-Plus HD, Ir-Plus 110°) had a trigger time ≤ 1 s and were furnished with external batteries (6 or 12 Volt) and 16–32 GB SD cards. The SD cards and batteries were replaced every c. 15–30 days and cameras were set to record videos of 10–30 s with no lag between one video and the next one. Cameras were set to work 24 h per day with “medium” PIR sensitivity. The sampling effort at each location was determined by the number of days between installation and checkout of the camera. When the batteries expired before the check period, the time of the last exposure was determined from the downloaded videos and considered as the last operational date [81]. At each camera trap site, we collected data on the following variables that could influence species detection, to account for them in statistical models [40]: 1) height of camera traps from the ground, 2) the percentage shrub cover (i.e., the vegetation up to 150 cm in height estimated visually in a 10-m radius around the camera trapping site); 3) the percentage canopy cover (i.e., trees over 150 cm in height on 10 m-radius circle around the cameras). For points 2 and 3 the surveys were carried out in March 2021. From each video, we obtained the following information: date, solar time, species, camera location. Operators who watched the videos and recognised the species first passed an accuracy test on 100 videos: they were allowed to participate in the identification procedure only when they correctly classified at least 95% videos.

### Temporal interactions: data analyses

When the same camera trap took more than one video of the same species within less than 30 min, we counted them as one event and considered the time of the first detection for analyses [23, 52, 93, 95]. To evaluate the potential influence of human activity on temporal and spatial relationships between the wolf and mesocarnivores, we also considered the frequency of human activity. To define human detections, we set a three-minute threshold between consecutive videos, after which we assessed whether they were different people/groups. The cumulative total sampling effort was 19,256 days with cameras working (*N* = 2665 wolf detections; 8090 red fox detections; 1242 badger detections; 811 *Martes* spp. detections, 14,322 human detections; Fig. 2; Additional file 2). In 1.8% human detections, related to the check of camera traps, the camera was not activated by the operator and the time of the detection was not recorded. Thus, we could not use those detections for temporal analyses. Temporal activity patterns of each species were assessed at the seasonal level (spring: April–June; summer: July–September; autumn: October–December; winter: January–March), using Kernel density estimation through the R package ‘overlap’ [57], and their 0.95 bootstrapped confidence intervals were estimated through 1000 replicates. Watson’s two-sample tests of homogeneity were calculated to compare activity patterns among carnivores and between carnivores and humans [53]. Interspecific overlap of temporal activity patterns was estimated according to Ridout and Linkie [77]. Among all species pairs, we calculated the non-parametric overlap coefficient (Δ: [98], values: 0–1 range, [48]). We adopted the Δ_4_ or the Δ_1_ coefficient depending on sample size ([77], see Additional file 3). For each species pair, we calculated 0.95 confidence intervals for overlap coefficients as percentile intervals from 1000 bootstrap samples [57]. We conducted temporal analyses for each year separately (Additional file 2–3) and for pooled years: results were consistent across years: we present pooled years in the main text.

High human activity may lead carnivores to concentrate their activity at night, thus emphasizing interspecific overlap with other carnivore species. To evaluate whether the interspecific temporal overlap between the wolf and mesocarnivores was influenced by human activity, we conducted a preliminary analysis by comparing overlap coefficients between sites with high human activity and sites with low human activity, separately [69]. We calculated the mean human detection rate across locations (i.e., number of detections over sampling effort defined as the number of days with camera working) and defined as “high human sites” those with human detection rate ≥ the mean value, and “low human sites” those with human detection rate < mean [63, 69, 80]. Although these analyses revealed a general slight increase in diurnal activity of our study species in “low human” sites than in “high human” ones (especially wolf and red fox), interspecific temporal overlap was generally high and comparable between sites (for details see Additional file 4). Thus, we included in the main text the analyses with no splitting in “high human” and “low human” sites. Similarly, we evaluated the temporal overlap between mesocarnivores and the wolf in sites with high and low activity of the apex predator [63, 80]. We used wolf detection rates to separate sites with high wolf activity (i.e., “high wolf” sites) from sites with low wolf activity (i.e., “low wolf” sites: Additional file 5). We used the Watson’s-two samples test to compare temporal activity patterns of mesocarnivores between “high wolf” and “low wolf” sites, and we calculated coefficients of interspecific temporal overlap separately for “high wolf” and “low wolf” sites (Additional file 5).

### Spatial interactions at a coarse temporal scale: data analyses

To evaluate the spatial association between wolf and mesocarnivore detection rates at a “coarse” temporal scale, for each location we estimated the monthly detection rate of our study species as the ratio of the number of detections over the number of operational days of cameras. We also considered the rate of human activity, to evaluate its potential effect on spatial variation of detection rates of wolves and mesocarnivores. Out of 1032 potential camera-months, we reported 154 months with no data because of battery failure, camera malfunctioning or theft (14.9%), and 19 months with data loss on people detections, which were excluded from these analyses. In one location used in the second and in the third year, the camera was deployed at a height of 2.9 m, to prevent the risk of theft because of logistical constraints. In 16 trapping months, this camera obtained only eight detections of red fox, one detection of badger and no *Martes* spp. detection, as well as 38 wolf detections (i.e., 1.4% of total wolf detections). Considering the potential bias towards the detection of large mammals determined by the height of the camera, we removed it from the analyses. Eventually, analyses of spatial interactions were based on 843 camera-months.

We evaluated whether monthly spatial variation of mesocarnivore detection rates were associated with wolf frequency of activity. We used generalised linear mixed models with negative binomial errors [103]. We fitted global models where the number of detections of the focal species in each sampling month was the response variable. The log (number of camera operating days) was included as offset variable to standardise the number of detections of our focal species for the actual sampling effort. Camera trapping location ID and camera model (IR-plus HD2, IR-plus 110; ScoutGuard; Comitel Guard1; Comitel Micro-Guard 2) were fitted as random effects, to account for potential effects of different camera trap models. Preliminary models tested for effects of shrub cover, canopy cover and camera height on carnivore detection rates; these models found some support for a positive effect of shrub cover on badger detection rate, as well as a positive effect of canopy cover on *Martes* detection rate (Additional file 6). Thus, we included shrub cover as a predictor in badger global models, and canopy cover in *Martes* global models. We also verified that differences in sampling effort among cameras did not affect detection rates of our focal species (Additional file 6). Eventually, we built a global model for each mesocarnivore, where we included the following variables as biologically plausible predictors: *i*) wolf monthly detection rate; *ii*) monthly detection rates of the other mesocarnivore species; *iii*) people monthly detection rate; *iv*) habitat (oakwood; pinewood; shrubwood; ecotone/meadows); *v*) season (spring: April-June; summer: July–September; autumn: October–December; winter: January–March); *vi*) study year (first: October 2017–September 2018; second: April 2019–March 2020; third: April 2020–March 2021); *vii*) percentage shrub cover (for badger models); *viii*) percentage canopy cover (*Martes* spp. models). The rationale for including these variables as predictors is shown in Additional file 6. Absence of collinearity among linear predictors was checked: pairs of predictors included in models did not show correlation coefficients >|0.6| (Additional file 6).

For each model set, we fitted candidate models including all potential different combinations of predictors, including also the null model, because each of them could represent different a priori hypotheses. The model selection used Akaike’s Information Criterion corrected for small sample sizes (AICc) and models were selected if they had AICc ≤ 2, and if their AICc value was lower than that of any simpler alternative [11]. Standardised model weight was calculated among selected models. Model selection was conducted through the R package ‘MuMIn’ [3]. We estimated the parameters (B coefficients and 95% confidence intervals) of the best models using the R packages ‘glmmTMB’ [9]. Best models were validated through visual inspection of residuals through the ‘DHARMa’ package [38]. For the red fox, three models were selected; and the badger study species, only the best model was selected; for *Martes* spp., two models were selected (Table 1).

### Finer-scale spatiotemporal interactions

To evaluate spatiotemporal interactions, we followed the method recommended by Niedballa et al. [68]. First, we considered each species pair separately, and defined each of them as “species A” or “species B”. Then, for each location separately, we calculated the temporal distance between each detection of species A and the next closest detection of species B, and the reverse. Thus, we defined as “AB” the temporal distances between detections of species A and the next detections of species B, and as “BA” the opposite. According to Niedballa et al. [68], we assumed that, if species B avoided the encounters with species A, we would have that AB > BA. We tested it through linear mixed models. For each species pair, we considered all the AB and BA temporal distances (log-transformed) as response variable,the type of distance (i.e., AB and BA) was fitted as a predictor to compare the time intervals AB with BA, with a positive effect indicating avoidance and a negative effect indicating attraction [68]. The camera location was added as random effect. We used only the series where no other species passage occurred in between the detections of species A and B, to properly evaluate the spatiotemporal interactions between the two target species. We used the same R packages described in *Spatial interactions: data analyses,* to estimate the parameters and validate the models.

## Supplementary Information

**Additional file 1**: Mesocarnivores in wolf diet.

**Additional file 2**: Temporal activity patterns of the wolf, mesocarnivores, and humans in single years.

**Additional file 3**: Interspecific temporal overlapin single years.

**Additional file 4**: Interspecific temporal overlapin sites with high vs. low human activity.

**Additional file 5**: Interspecific temporal overlapin sites with high vs. low wolf activity.

**Additional file 6**: Preliminary analyses on spatial relationships between the wolf and mesocarnivores.

**Additional file 7**: Wolf-mesocarnivore spatial relationships in the same area sampled in the first study year.

## Data Availability

The datasets used and/or analysed during the current study have been collected under an agreement between the Maremma Regional Park Agency and the Department of Life Sciences – University of Siena – and are available from the corresponding author on reasonable request and upon permission of the above mentioned parties.

## References

[1] Allen ML, Elbroch LM, Wilmers CC, Wittmer HU (2015). The comparative effects of large carnivores on the acquisition of carrion by scavengers. Am Nat.

[2] Balestrieri A, Remonti L, Ruiz-González A, Vergara M, Capelli E, Gómez B, Prigioni C (2011). Food habits of genetically identified pine marten (*Martes martes*) expanding in agricultural lowlands (NW Italy). Acta Theriol.

[3] Bartoń K. MuMIn: multi-model inference. R package version 1.15.6. 2012;Available from URL: https://cran.r-project.org/web/packages/MuMIn.

[4] Bassi E, Donaggio E, Marcon A, Scandura M, Apollonio M (2012). Trophic niche overlap and wild ungulate consumption by red fox and wolf in a mountain area in Italy. Mamm Biol.

[5] Bauder JM, Allen ML, Ahlers AA, Benson TJ, Miller CA, Stodola KW (2022). Long-term data reveal equivocal evidence for intraguild suppression among sympatric canids. Biodivers Conserv.

[6] Birks J, Messenger J, Halliwell E (2005). Diversity of den sites used by pine martens *Martes martes*: a response to the scarcity of arboreal cavities?. Mamm Rev.

[7] de Boer WD, Prins HH (1990). Large herbivores that strive mightily but eat and drink as friends. Oecologia.

[8] Broekhuis F, Cozzi G, Valeix M, McNutt JW, Macdonald DW (2013). Risk avoidance in sympatric large carnivores: reactive or predictive?. J Anim Ecol.

[9] Brooks ME, Kristensen K, van Benthem KJ, Magnusson A, Berg CW, Nielsen A, Skaug HJ, Mächler MM, Bolker BM (2017). GlmmTMB balances speed and flexibility among packages for zero-inflated generalized linear mixed modelling. R J.

[10] Bruno JF, Stachowicz JJ, Bertness MD (2003). Inclusion of facilitation into ecological theory. Trends Ecol Evol.

[11] Burnham KP, Anderson DR (2002). Model selection and multimodel inference: a practical information-theoretic approach.

[12] Carbone C, Pettorelli N, Stephens PA (2010). The bigger they come, the harder they fall: body size and prey abundance influence predator-prey ratios. Biol Lett.

[13] Cavallini P, Lovari S (1991). Environmental factors influencing the use of habitat in the red fox. Vulpes vulpes J Zool.

[14] Cavallini P, Lovari S (1994). Home range, habitat selection and activity of the red fox in a Mediterranean coastal ecotone. Acta Theriol.

[15] Chapron G, Kaczensky P, Linnell JDC, von Arx M, Huber D, Andrén H (2014). Recovery of large carnivores in Europe’s modern human-dominated landscapes. Science.

[16] Ciampalini B, Lovari S (1985). Food habits and trophic niche overlap of the badger (*Meles meles* L.) and the red fox (*Vulpes vulpes* L.) in a Mediterranean coastal area. Z Saugetierkd..

[17] Ciucci P, Boitani L, Pelliccioni ER, Rocco M, Guy I (1996). A comparison of scat‐analysis methods to assess the diet of the wolf *Canis lupus*. Wildl Biol.

[18] Ciucci P, Boitani L, Francisci F, Andreoli G (1997). Home range, activity and movements of a wolf pack in central Italy. J Zool.

[19] Crooks K, Soulé M (1999). Mesopredator release and avifaunal extinctions in a fragmented system. Nature.

[20] Cusack JJ, Kohl MT, Metz MC, Coulson T, Stahler DR, Smith DW, MacNulty DR (2019). Weak spatiotemporal response of prey to predation risk in a freely interacting system. J Anim Ecol.

[21] Davis ML, Stephens PA, Willis SG, Bassi E, Marcon A, Donaggio E, Capitani C, Apollonio M (2012). Prey selection by an apex predator: the importance of sampling uncertainty. PLoS ONE.

[22] De Marinis AM, Asprea A (2006). Hair identification key of wild and domestic ungulates from southern Europe. Wildl Biol.

[23] de Satgé J, Teichman K, Cristescu B (2017). Competition and coexistence in a small carnivore guild. Oecologia.

[24] Díaz-Ruiz F, Caro J, Delibes-Mateos M, Arroyo B, Ferreras P (2016). Drivers of red fox (*Vulpes vulpes*) daily activity: prey availability, human disturbance or habitat structure?. J Zool.

[25] Diserens T, Bubnicki J, Schutgens E, Rokx K, Kowalczyk R, Kuijper DPJ, Churski M (2020). Fossoriality in a risky landscape: badger sett use varies with perceived wolf risk. J Zool.

[26] Diserens TA, Churski M, Bubnicki JW, Zalewski A, Brzeziński M, Kuijper DP (2022). Wolf risk fails to inspire fear in two mesocarnivores suggesting facilitation prevails. Sci Rep.

[27] Donadio E, Buskirk S (2006). Diet, morphology, and interspecific killing in Carnivora. Am Nat.

[28] Durant SM (1998). Competition refuges and coexistence: an example from Serengeti carnivores. J Anim Ecol.

[29] Esattore B, Rossi A, Bazzoni F, Riggio C, Oliveira R, Leggiero I, Ferretti F (2023). Same place, different time, head up: multiple antipredator responses to a recolonising apex predator. Curr Zool.

[30] Estes JA, Terborgh J, Brashares JS, Power ME, Berger J, Bond WJ (2011). Trophic downgrading of planet earth. Science.

[31] Fattorini N, Ferretti F (2020). Estimating wild boar density and rooting activity in a Mediterranean protected area. Mamm Biol.

[32] Ferreiro-Arias AI, Isla J, Jordano P, Benítez-López A (2021). Fine-scale coexistence between Mediterranean mesocarnivores is mediated by spatial, temporal, and trophic resource partitioning. Ecol Evol.

[33] Ferretti F, Fattorini N (2021). Competitor densities, habitat, and weather: effects on interspecific interactions between wild deer species. Integr Zool.

[34] Ferretti F, Lovari S, Mancino V, Burrini L, Rossa M (2019). Food habits of wolves and selection of wild ungulates in a prey-rich Mediterranean coastal area. Mamm Biol.

[35] Ferretti F, Lovari S, Lucherini M, Hayward M, Stephens PA (2020). Only the largest terrestrial carnivores increase their dietary breadth with increasing prey richness. Mamm Rev.

[36] Ferretti F, Pacini G, Belardi I, Ten Cate B, Sensi M, Oliveira R, Rossa M, Burrini L, Lovari S (2021). Recolonizing wolves and opportunistic foxes: interference or facilitation?. Biol J Linn Soc.

[37] Gaynor K, Hojnowski C, Neil C, Brashares J (2018). The influence of human disturbance on wildlife nocturnality. Science.

[38] Hartig F. DHARMa: residual diagnostics for hierarchical (multi-level/mixed) regression models. 2022. https://cran.r-project.org/web/packages/DHARMa/vignettes/DHARMa.html

[39] Haswell PM, Kusak J, Hayward MW (2017). Large carnivore impacts are context-dependent. Food Webs.

[40] Hofmeester T, Cromsigt J, Odden J, Andrén H, Kindberg J, Linnell J (2019). Framing pictures: a conceptual framework to identify and correct for biases in detection probability of camera traps enabling multi-species comparison. Ecol Evol.

[41] Hunter J, Durant S, Caro T (2007). To flee or not to flee: predator avoidance by cheetahs at kills. Behav Ecol Sociobiol.

[42] Jachowski DS, Butler A, Eng RYY, Gigliotti L, Harris S, Williams A (2020). Identifying mesopredator release in multi-predator systems: a review of evidence from North America. Mamm Rev.

[43] Karanth KU, Srivathsa A, Vasudev D, Puri M, Parameshwaran R, Kumar NS (2017). Spatio-temporal interactions facilitate large carnivore sympatry across a resource gradient. Proc R Soc B.

[44] Khan U, Lovari S, Ali Shah S, Ferretti F (2018). Predator, prey and humans in a mountainous area: loss of biological diversity leads to trouble. Biodivers Conserv.

[45] Kowalczyk R, Jȩdrzejewska B, Zalewski A (2003). Annual and circadian activity patterns of badgers (*Meles meles*) in Białowieża Primeval Forest (eastern Poland) compared with other Palaearctic populations. J Biogeogr.

[46] Kuijper DPJ, Sahlén E, Elmhagen B, Chamaillé-Jammes S, Sand H, Lone K, Cromsigt JPGM (2016). Paws without claws? Ecological effects of large carnivores in anthropogenic landscapes. Proc R Soc B.

[47] Leo V, Reading RP, Letnic M (2015). Interference competition: odours of an apex predator and conspecifics influence resource acquisition by red foxes. Oecologia.

[48] Linkie M, Ridout MS (2011). Assessing tiger-prey interactions in Sumatran rainforests. J Zool.

[49] Lovari S, Valier P, Ricci Lucchi M (1994). Ranging behaviour and activity of red foxes (*Vulpes vulpes*: Mammalia) in relation to environmental variables, in a Mediterranean mixed pinewood. J Zool.

[50] Lovari S, Boesi R, Minder I, Mucci N, Randi E, Dematteis A, Ale S (2009). Restoring a keystone predator may endanger a prey species in a human-altered ecosystem: the return of the snow leopard to Sagarmatha National Park. Anima Conserv.

[51] Lovari S, Pokheral CP, Jnawali SR, Fusani L, Ferretti F (2015). Coexistence of the tiger and the common leopard in a prey-rich area: the role of prey partitioning. J Zool.

[52] Lucherini M, Reppucci JI, Walker RS, Villalba ML, Wurstten A, Gallardo G, Iriarte A, Villalobos R, Perovic P (2009). Activity pattern segregation of carnivores in the High Andes. J Mammal.

[53] Lund U, Agostinelli C, Arai H, Gagliardi A, Portugues EG, Giunchi D, Irisson JO, Pocernich M, Rotolo F. Circular Statistics. 2017. https://cran.r-project.org/web/packages/circular/circular.

[54] Martinoli A, Preatoni D (1995). Food habits of the stone marten (*Martes foina*) in the upper Aveto Valley (Northern Apennines, Italy). Hystrix It J Mammal.

[55] Mattioli L, Capitani C, Gazzola A, Scandura M, Apollonio M (2011). Prey selection and dietary response by wolves in a high-density multi-species ungulate community. Eur J Wildl Res.

[56] Melini D, Agrillo E, Ferretti F, Tonelli L. Piano di Gestione della ZSC/ZPS IT51A0016 Monti dell’Uccellina. Alberese: Ente Parco Regionale della Maremma; 2019.

[57] Meredith M, Ridout M. Overlap: estimates of coefficient of overlapping for animal activity patterns. 2017. https://cran.r-project.org/web/packages/overlap/overlap.pdf.

[58] Mergey M, Helder R, Roeder JJ (2011). Effect of forest fragmentation on space-use patterns in the European pine marten (*Martes martes*). J Mammal.

[59] Meriggi A, Brangi A, Schenone L, Signorelli D, Milanesi P (2011). Changes of wolf (*Canis lupus*) diet in Italy in relation to the increase of wild ungulate abundance. Ethol Ecol Evol.

[60] Meriggi A, Lovari S (1996). A review of wolf predation in southern Europe: does the wolf prefer wild prey to livestock?. J Appl Ecol.

[61] Monterroso P, Alves PC, Ferreras P (2013). Catch me if you can: diel activity patterns of mammalian prey and predators. Ethology.

[62] Monterroso P, Alves PC, Ferreras P (2014). Plasticity in circadian activity patterns of mesocarnivores in southwestern Europe: implications for species coexistence. Behav Ecol Sociobiol.

[63] Mori E, Bagnato S, Serroni P, Sangiuliano A, Rotondaro F, Marchianò V, Cascini V, Poerio L, Ferretti F (2020). Spatiotemporal mechanisms of coexistence in a European mammal community in a protected area of southern Italy. J Zool.

[64] Mori E, Fedele E, Greco I, Rustichelli M, Massolo A, Miniati S, Puppo F, Santini G, Zaccaroni M (2021). Spatiotemporal activity of the pine marten *Martes martes*: insights from an island population. Ecol Res.

[65] Mori E, Menchetti M (2019). Living with roommates in a shared den: spatial and temporal segregation among semifossorial mammals. Behav Process.

[66] Nelson AA, Kauffman MJ, Middleton A, Jimenez M, McWhirter D, Barber J, Gerow K (2012). Elk migration patterns and human activity influence wolf habitat use in the Greater Yellowstone Ecosystem. Ecol Appl.

[67] Newsome T, Boitani L, Chapron G, Ciucci P, Dickman C, Dellinger J, López-Bao JV, Peterson R, Shores C, Wirsing A, Ripple W (2016). Food habits of the world's grey wolves. Mam Rev.

[68] Niedballa J, Wilting A, Sollmann R, Hofer H, Courtiol A (2019). Assessing analytical methods for detecting spatiotemporal interactions between species from camera trapping data. Remote Sens Ecol Conserv.

[69] Oberosler V, Groff C, Iemma A, Pedrini P, Rovero F (2017). The influence of human disturbance on occupancy and activity patterns of mammals in the Italian Alps from systematic camera trapping. Mamm Biol.

[70] Palomares F, Caro T (1999). Interspecific killing among mammalian carnivores. Am Nat.

[71] Palomares F, Ferreras P, Fedriani JM, Delibes M (1996). Spatial relationships between Iberian lynx and other carnivores in an area of south-western Spain. J Appl Ecol.

[72] Pasanen-Mortensen M, Pyykönen M, Elmhagen B (2013). Where lynx prevail, foxes will fail—limitation of a mesopredator in Eurasia. Glob Ecol Biogeogr.

[73] Patalano M, Lovari S (1993). Food habits and trophic niche overlap of the wolf *Canis lupus*, L. 1758 and the red fox *Vulpes vulpes* (L. 1758) in a Mediterranean mountain area. Revue d’Ecologie (Terre Vie).

[74] Pilot M, Gralak B, Goszczyński J, Posluszny M (2007). A method of genetic identification of pine marten (*Martes martes*) and stone marten (*Martes foina*) and its application to faecal samples. J Zool.

[75] Prugh LR, Sivy JK (2020). Enemies with benefits: integrating positive and negative interactions among terrestrial carnivores. Ecol Lett.

[76] Richards DF (1977). Observations on the diet of the Red fox (*Vulpes vulpes*) in South Devon. J Zool.

[77] Ridout MS, Linkie M (2009). Estimating overlap of daily activity patterns from camera trap data. J Agric Biol Environ Stat.

[78] Ripple WJ, Estes JA, Beschta RL, Wilmers CC, Ritchie EG, Hebblewhite M (2014). Status and ecological effects of the world’s largest carnivores. Science.

[79] Rosellini S, Osorio E, Ruiz-González A, Piñeiro A, Barja I (2008). Monitoring the small-scale distribution of sympatric European pine martens (*Martes martes*) and stone martens (*Martes foina*): a multievidence approach using faecal DNA analysis and camera-traps. Wildl Res.

[80] Rossa M, Lovari S, Ferretti F (2021). Spatiotemporal patterns of wolf, mesocarnivores and prey in a Mediterranean area. Behav Ecol Sociobiol.

[81] Rowcliffe JM, Field J, Turvey ST, Carbone C (2008). Estimating animal density using camera traps without the need for individual recognition. J Appl Ecol.

[82] Ruprecht J, Eriksson CE, Forrester TD, Spitz DB, Clark DA, Wisdom MJ, Bianco M, Rowland MM, Smith JB, Johnson BK, Levi T (2021). Variable strategies to solve risk–reward trade-offs in carnivore communities. PNAS.

[83] Salvatori M, Oberosler V, Rinaldi M, Franceschini A, Truschi S, Pedrini P, Rovero F. Crowded mountains: Long-term effects of human outdoor recreation on a community of wild mammals monitored with systematic camera trapping. Ambio. 2023. 1–13. 10.1007/s13280-022-01825-w 10.1007/s13280-022-01825-w PMC10160289 36626062

[84] Selva N, Fortuna MA (2007). The nested structure of a scavenger community. Proc R Soc B.

[85] Selva N, Jedrzejewska B, Jedrzejewski W, Wajrak A (2005). Factors affecting carcass use by a guild of scavengers in European temperate woodland. Can J Zool.

[86] Serafini P, Lovari S (1993). Food habits and trophic niche overlap of the red fox and the stone marten in a Mediterranean rural area. Acta Theriol.

[87] Sforzi A, Tonelli L, Selva FC, Mastacchi R, Lanzi L, Anselmi G, Martini G, Naviglio L. Piano di Gestione dei SIC/SIR - IT51A0039 [SIE 113 e A113(ZPS)] Palude della Trappola e Bocca d’Ombrone, IT51A0014 [SIR 114] Pineta Granducale dell’Uccellina e IT51A0015 [SIR 115] Dune costiere del Parco dell’Uccellina. Ente Parco Regionale della Maremma, Alberese. 2012.

[88] Sivy KJ, Pozzanghera CB, Colson KE, Mumma MA, Prugh LR (2018). Apex predators and the facilitation of resource partitioning among mesopredators. Oikos.

[89] Stachowicz JJ (2001). Mutualism, facilitation, and the structure of ecological communities. Bioscience.

[90] Teerink BJ (1991). Hair of West European Mammals: atlas and identification key.

[91] Theuerkauf J (2009). What drives wolves: fear or hunger? Humans, diet, climate and wolf activity patterns. Ethology.

[92] Theuerkauf J, Jedrzejewski W, Schmidt K, Okarma H, Ruczyński I, Śnieżko S, Roman G (2003). Daily patterns and duration of wolf activity in the Białowieża Forest, Poland. J Mammal.

[93] Tobler MW, Carrillo-Percastegui SE, Leite Pitman R, Mares R, Powell G (2008). An evaluation of camera traps for inventorying large- and medium-sized terrestrial rainforest mammals. Anim Conserv.

[94] Torretta E, Mosini A, Piana M, Tirozzi P, Serafini M, Puopolo F, Saino N, Balestrieri A (2017). Time partitioning in mesocarnivore communities from different habitats of NW Italy: insights into martens' competitive abilities. Behaviour.

[95] Torretta E, Serafini M, Puopolo F, Schenone L (2016). Spatial and temporal adjustments allowing the coexistence among carnivores in Liguria (N-W Italy). Acta Ethol.

[96] Vanak AT, Fortin D, Thaker M, Ogden M, Owen C, Greatwood S, Slotow R (2013). Moving to stay in place: behavioral mechanisms for coexistence of African large carnivores. Ecology.

[97] Wearn O, Glover-Kapfer P. Camera-trapping for conservation: a guide to best-practices. Woking: WWF conservation technology series 1. WWF-UK; 2017. 10.13140/RG.2.2.23409.17767.

[98] Weitzman MS. Measure of overlap of income distributions of white and negro families in the United States. Washington: US Government Printing Office; 1970. USA.

[99] Wikenros C, Aronsson M, Liberg O, Jarnemo A, Hansson J, Wallgren M, Sand H, Bergström R (2017). Fear or food—abundance of red fox in relation to occurrence of lynx and wolf. Sci Rep.

[100] Wikenros C, Kuijper DP, Behnke R, Schmidt K (2015). Behavioural responses of ungulates to indirect cues of an ambush predator. Behaviour.

[101] Wikenros C, Sand H, Ahlqvist P, Liberg O (2013). Biomass flow and scavengers use of carcasses after re-colonization of an apex predator. PLoS ONE.

[102] Wilson EE, Wolkovich EM (2011). Scavenging: how carnivores and carrion structure communities. Trends Ecol Evol.

[103] Zuur AF, Ieno EN, Walker NJ, Saveliev AA, Smith GM (2009). Mixed effects models and extensions in ecology with R.

