# Supplementary material for: Interactions between carnivore species: limited spatiotemporal partitioning between apex predator and smaller carnivores in a Mediterranean protected area
Source: Front Zool. 2023 May 25;20:20. doi: 10.1186/s12983-023-00489-w (PMC10210480; doi:10.1186/s12983-023-00489-w)
Supplement: Supplementary file 1 — Additional file 1: Mesocarnivores in wolf diet. [file 12983_2023_489_MOESM1_ESM.docx]

**Title:** Interactions between carnivore species: limited spatiotemporal partitioning between apex predator and smaller carnivores in a Mediterranean protected area

**Author list:** Francesco Ferretti^1,2*^, Raquel Oliveira^1^, Mariana Rossa^3^, Irene Belardi^1^, Giada Pacini^1^, Sara Mugnai^1^, Niccolò Fattorini^1^ & Lorenzo Lazzeri^1^

**Affiliations:** ^1^Research Unit of Behavioural Ecology, Ethology and Wildlife Management – Department of Life Sciences – University of Siena. Via P.A. Mattioli 4, 53100, Siena, Italy; ^2^NBFC, National Biodiversity Future Center, Palermo 90133, Italy; ^3^CESAM, Department of Biology, University of Aveiro, Campus de Santiago, 3810-193 Aveiro, Portugal

**Corresponding author:** Francesco Ferretti, Research Unit of Behavioural Ecology, Ethology and Wildlife Management – Department of Life Sciences – University of Siena. Via P.A. Mattioli 4, 53100, Siena, Italy. E-mail: [francesco.ferretti@unisi.it](about:blank).

**Additional file 1**

*Mesocarnivores in wolf diet*

We assessed the frequency of occurrence of mesocarnivores in the wolf diet by analysing wolf food habits through the identification of indigested remains in scats (Ciucci et al. 1996; Ferretti et al. 2019, 2021, for our study area). Wolf scats were collected monthly (April 2016-March 2021) along itineraries for a total of up to *c.* 120 km/month, and opportunistically during usual activities of territory patrolling by Park Wardens. The scat identification in the field was based on a set of characteristics such as size, texture, shape, content, position, and their characteristic odor (Lovari et al. 2009). Dubious scats were not collected; no free-ranging/feral dogs were recorded by camera traps in the area. DNA genotyping of a subsample of fresh putative wolf scats (*N* = 55; Istituto Superiore per la Protezione e la Ricerca Ambientale, unpublished; Fazzi et al. 2018) confirmed species identification, supporting our field criteria. Samples were collected using a polythene bag; scats collected in the same place, at the same time, and showing the same apparent age and content were not considered for analyses. Samples were stored in the freezer (-20°C), after which they were analyzed in the laboratory (Cavallini and Lovari 1991; Ferretti et al. 2019).

Overall, 2,201 scats were collected and used for analyses (April 2016-March 2017: *n* = 72; April 2017-March 2018: *n* = 347; April 2018-March 2019: *n* = 589; April 2019-March 2020: *n* = 594; April 2020-March 2021: *n* = 599). Each sample was dried in an oven at 80° C for over 4 hours to inactivate potential parasites harmful to humans. Then, the content of each scat was assessed according to Lovari et al. (2009, 2015). Samples were examined using tweezers, then washed in water through a 1-3 mm mesh sieve (Kruuk and Parish, 1981) to separate the undigested parts such as hair, fragments of bones, teeth, quills, hooves, fragments of plants or unidentified constituents. These macro constituents, useful for the identification of the prey, were washed, dried, and stored for further analyses. The identification of hairs was made through (*i*) a macroscopic comparison of hairs with a reference collection of hairs of potential wolf prey, using parameters such as color, shape, length, and thickness, and (*ii*) microscopic analyses of cuticle, medulla, and cortex, under an optical microscope (100-400x), through which hair features were compared with reference atlases, identification keys and reference collection of hair of local prey (Teerink 1991; De Marinis and Asprea 2006). A blind test was performed to test the skills of the observer (G.P., I.B. and M.R) in scat analyses: a sample of 75 slides and 25 bags including reference hair were shown to each observer for identification. Observers were allowed to conduct analyses when they identified ≥ 95% of reference samples correctly. For the purposes of this work, wolf prey was categorised as “large herbivores” (i.e., wild boar, fallow deer, roe deer, or livestock), “red fox”, “badger”, “*Martes* spp.”, “other mammals”, “other vertebrates”, “invertebrates”, “fruits” and “unidentified” items. For each *i-*th category, we calculated its frequency of occurrence in the diet as the percentage of scats including it. Then, we calculated bootstrap 0.95 confidence intervals of frequency of occurrence through 1000 replicates.

**References**

Cavallini P, Lovari S. Environmental factors influencing the use of habitat in the red fox *Vulpes vulpes* J Zool. 1991;223:323–39.

Ciucci P, Boitani L, Pelliccioni ER, Rocco M, Guy I. A comparison of scat‐analysis methods to assess the diet of the wolf *Canis lupus*. Wildl Biol. 1996; 2:37-48

De Marinis AM, Asprea A. Hair identification key of wild and domestic ungulates from southern Europe. Wildl Biol 2006;12:305–20.

Fazzi P, Lucchesi M, Ricci S, Salvatori V (2018) Indagine sulla presenza del lupo in Provincia di Grosseto. Relazione tecnica delle attività svolte nel Parco Regionale della Maremma. Progetto LIFE MEDWOLF, Rome, Italy: Istituto di Ecologia Applicata. [In Italian]

Ferretti F, Lovari S, Mancino V, Burrini L, Rossa M. Food habits of wolves and selection of wild ungulates in a prey-rich Mediterranean coastal area. Mamm Biol. 2019;99:119–27.

Ferretti F, Pacini G, Belardi I, Ten Cate B, Sensi M, Oliveira R, Rossa M, Burrini L, Lovari S. Recolonizing wolves and opportunistic foxes: interference or facilitation? Biol J Linn Soc. 2021;132:196–210

Kruuk H, Parish T. Feeding specialization of the European badger *Meles meles* in Scotland. J Anim Ecol. 1981; 50:773–88.

Lovari S, Boesi R, Minder I, Mucci N, Randi E, Dematteis A, Ale SB. Restoring a keystone predator may endanger a prey species in a human‐altered ecosystem: the return of the snow leopard to Sagarmatha National Park. Anim Conserv. 2009; 12:559-570.

Teerink BJ. Hair of West European Mammals: atlas and identification key. Cambridge: Cambridge University Press; 1991.
