# Supplementary material for: Interactions between carnivore species: limited spatiotemporal partitioning between apex predator and smaller carnivores in a Mediterranean protected area
Source: Front Zool. 2023 May 25;20:20. doi: 10.1186/s12983-023-00489-w (PMC10210480; doi:10.1186/s12983-023-00489-w)
Supplement: Supplementary file 2 — Additional file 2: Temporal activity patterns of the wolf, mesocarnivores, and humans in single years. [file 12983_2023_489_MOESM2_ESM.docx]

**Title:** Interactions between carnivore species: limited spatiotemporal partitioning between apex predator and smaller carnivores in a Mediterranean protected area

**Author list:** Francesco Ferretti^1,2*^, Raquel Oliveira^1^, Mariana Rossa^3^, Irene Belardi^1^, Giada Pacini^1^, Sara Mugnai^1^, Niccolò Fattorini^1^ & Lorenzo Lazzeri^1^

**Affiliations:** ^1^Research Unit of Behavioural Ecology, Ethology and Wildlife Management – Department of Life Sciences – University of Siena. Via P.A. Mattioli 4, 53100, Siena, Italy; ^2^NBFC, National Biodiversity Future Center, Palermo 90133, Italy; ^3^CESAM, Department of Biology, University of Aveiro, Campus de Santiago, 3810-193 Aveiro, Portugal

**Corresponding author:** Francesco Ferretti, Research Unit of Behavioural Ecology, Ethology and Wildlife Management – Department of Life Sciences – University of Siena. Via P.A. Mattioli 4, 53100, Siena, Italy. E-mail: [francesco.ferretti@unisi.it](about:blank).

**Additional file 2**

Temporal activity patterns of wolf, red fox, badger, *Martes* spp., and humans, were estimated at the seasonal scale (spring: April-June; summer: July-September; autumn: October-December; winter: January-March), in the three different study years (First year: October 2017-September 2018; Second year: April 2019-March 2020; Third year: April 2020-March 2021) (Figures S1-S5). Temporal activity patterns were estimated through kernel density estimators, together with bootstrapped 0.95 confidence intervals. For a detailed description of methods, see main text.

**Figure S1.** Temporal activity patterns of the wolf at seasonal scale (spring: April-June; summer: July-September; autumn: October-December; winter: January-March) in the three study years (First year: October 2017-September 2018; Second year: April 2019-March 2020; Third year: April 2020-March 2021). Red solid lines indicate estimates of temporal activity patterns through kernel density estimators; colored lines represent bootstrapped estimates of activity patterns through 1000 replicates; dashed red lines represent 0.95 bootstrapped confidence intervals. Grey rectangles indicate times of day of dawn and dusk during each season; black rectangles indicate times of day preceding the dawn and following the dusk. Sample size (i.e., number of detections) is shown in each panel.

**Figure S2.** Temporal activity patterns of the red fox at seasonal scale (spring: April-June; summer: July-September; autumn: October-December; winter: January-March) in the three study years (First year: October 2017-September 2018; Second year: April 2019-March 2020; Third year: April 2020-March 2021). Red solid lines indicate estimates of temporal activity patterns through kernel density estimators; colored lines represent bootstrapped estimates of activity patterns through 1000 replicates; dashed red lines represent 0.95 bootstrapped confidence intervals. Grey rectangles indicate times of day of dawn and dusk during each season; black rectangles indicate times of day preceding the dawn and following the dusk. Sample size (i.e., number of detections) is shown in each panel.

**Figure S3.** Temporal activity patterns of the badger at seasonal scale (spring: April-June; summer: July-September; autumn: October-December; winter: January-March) in the three study years (First year: October 2017-September 2018; Second year: April 2019-March 2020; Third year: April 2020-March 2021). Red solid lines indicate estimates of temporal activity patterns through kernel density estimators; colored lines represent bootstrapped estimates of activity patterns through 1000 replicates; dashed red lines represent 0.95 bootstrapped confidence intervals. Grey rectangles indicate times of day of dawn and dusk during each season; black rectangles indicate times of day preceding the dawn and following the dusk. Sample size (i.e., number of detections) is shown in each panel.

**Figure S4.** Temporal activity patterns of the *Martes* spp. at seasonal scale (spring: April-June; summer: July-September; autumn: October-December; winter: January-March) in the three study years (First year: October 2017-September 2018; Second year: April 2019-March 2020; Third year: April 2020-March 2021). Red solid lines indicate estimates of temporal activity patterns through kernel density estimators; colored lines represent bootstrapped estimates of activity patterns through 1000 replicates; dashed red lines represent 0.95 bootstrapped confidence intervals. Grey rectangles indicate times of day of dawn and dusk during each season; black rectangles indicate times of day preceding the dawn and following the dusk. Sample size (i.e., number of detections) is shown in each panel.

**Figure S5.** Temporal activity patterns of humans at seasonal scale (spring: April-June; summer: July-September; autumn: October-December; winter: January-March) in the three study years (First year: October 2017-September 2018; Second year: April 2019-March 2020; Third year: April 2020-March 2021). Red solid lines indicate estimates of temporal activity patterns through kernel density estimators; colored lines represent bootstrapped estimates of activity patterns through 1000 replicates; dashed red lines represent 0.95 bootstrapped confidence intervals. Grey rectangles indicate times of day of dawn and dusk during each season; black rectangles indicate times of day preceding the dawn and following the dusk. Sample size (i.e., number of detections) is shown in each panel.
