# Supplementary material for: Interactions between carnivore species: limited spatiotemporal partitioning between apex predator and smaller carnivores in a Mediterranean protected area
Source: Front Zool. 2023 May 25;20:20. doi: 10.1186/s12983-023-00489-w (PMC10210480; doi:10.1186/s12983-023-00489-w)
Supplement: Supplementary file 3 — Additional file 3: Interspecific temporal overlapin single years. [file 12983_2023_489_MOESM3_ESM.docx]

**Title:** Interactions between carnivore species: limited spatiotemporal partitioning between apex predator and smaller carnivores in a Mediterranean protected area

**Author list:** Francesco Ferretti^1,2*^, Raquel Oliveira^1^, Mariana Rossa^3^, Irene Belardi^1^, Giada Pacini^1^, Sara Mugnai^1^, Niccolò Fattorini^1^ & Lorenzo Lazzeri^1^

**Affiliations:** ^1^Research Unit of Behavioural Ecology, Ethology and Wildlife Management – Department of Life Sciences – University of Siena. Via P.A. Mattioli 4, 53100, Siena, Italy; ^2^NBFC, National Biodiversity Future Center, Palermo 90133, Italy; ^3^CESAM, Department of Biology, University of Aveiro, Campus de Santiago, 3810-193 Aveiro, Portugal

**Corresponding author:** Francesco Ferretti, Research Unit of Behavioural Ecology, Ethology and Wildlife Management – Department of Life Sciences – University of Siena. Via P.A. Mattioli 4, 53100, Siena, Italy. E-mail: [francesco.ferretti@unisi.it](about:blank).

**Additional file 3**

Temporal overlap between wolf and red fox, badger, and *Martes* spp., as well as between carnivores and humans, was estimated at the seasonal scale (spring: April-June; summer: July-September; autumn: October-December; winter: January-March), in the three different study years (First year: October 2017-September 2018; Second year: April 2019-March 2020; Third year: April 2020-March 2021) (Figures S6-S7). Coefficients of temporal overlap (∆_1_ or ∆_4_ coefficients, depending on sample size) and bootstrapped 0.95 confidence intervals were estimated. For a detailed description of methods, see main text.

**Figure S6.** Temporal overlap among wolf and mesocarnivores estimated through nonparametric coefficient of overlap (∆_1_ or ∆_4_ coefficients, depending on sample size), at the seasonal scale (2017-2021). Error bars indicate bootstrapped 0.95 confidence intervals obtained through 1000 replicates. For *Martes* in summer 2017-2018, only 8 detections were collected. * = *p* < 0.05; **: *p* < 0.01; ***: *p* < 0.001, two-sample Watson’s test.

**Figure S7.** Temporal overlap among humans and carnivores estimated through nonparametric coefficient of overlap (∆_1_ or ∆_4_ coefficients, depending on sample size), at the seasonal scale (2017-2021). Error bars indicate bootstrapped 0.95 confidence intervals obtained through 1000 replicates. For *Martes* in summer 2017-2018, only 8 detections were collected. Interspecific differences of temporal activity patterns were always significant (*U* = 0.408-76.832, *p* < 0.001), two-sample Watson’s test.
