# Supplementary material for: Interactions between carnivore species: limited spatiotemporal partitioning between apex predator and smaller carnivores in a Mediterranean protected area
Source: Front Zool. 2023 May 25;20:20. doi: 10.1186/s12983-023-00489-w (PMC10210480; doi:10.1186/s12983-023-00489-w)
Supplement: Supplementary file 4 — Additional file 4: Interspecific temporal overlapin sites with high vs. low human activity. [file 12983_2023_489_MOESM4_ESM.docx]

**Title:** Interactions between carnivore species: limited spatiotemporal partitioning between apex predator and smaller carnivores in a Mediterranean protected area

**Author list:** Francesco Ferretti^1,2*^, Raquel Oliveira^1^, Mariana Rossa^3^, Irene Belardi^1^, Giada Pacini^1^, Sara Mugnai^1^, Niccolò Fattorini^1^ & Lorenzo Lazzeri^1^

**Affiliations:** ^1^Research Unit of Behavioural Ecology, Ethology and Wildlife Management – Department of Life Sciences – University of Siena. Via P.A. Mattioli 4, 53100, Siena, Italy; ^2^NBFC, National Biodiversity Future Center, Palermo 90133, Italy; ^3^CESAM, Department of Biology, University of Aveiro, Campus de Santiago, 3810-193 Aveiro, Portugal

**Corresponding author:** Francesco Ferretti, Research Unit of Behavioural Ecology, Ethology and Wildlife Management – Department of Life Sciences – University of Siena. Via P.A. Mattioli 4, 53100, Siena, Italy. E-mail: [francesco.ferretti@unisi.it](about:blank).

**Additional file 4**

To evaluate whether interspecific temporal overlap between the wolf and mesocarnivores was influenced by human activity, we conducted a preliminary analysis by calculating overlap coefficients for sites with high human activity and sites with low human activity, separately (Oberosler et al. 2017; Mori et al. 2020; Rossa et al. 2021). For each yearly study period and for each location, we calculated the human detection rate as ratio of the number of human detections over the number of days with camera operating. Then, for each year we calculated the mean human detection rate across locations, and defined as “high human sites” those with human detection rate ≥ the mean value, and “low human sites” those with human detection rate < mean (Oberosler et al. 2017; Mori et al. 2020; Rossa et al. 2021). For these analyses, we did not consider 19 months with data loss on human detections. In this way, we defined as “high human” sites 3 locations in the first period, 14 locations in the second period, and 11 locations in the third period. Furthermore, we did not consider a location sampled in the second and third year, because the camera was deployed at a height of 2.9 m, to prevent the risk of theft because of logistical constraints. In 16 trapping months, this camera obtained only eight detections of red fox, one detection of badger and no *Martes* spp. detection, as well as 38 wolf detections (i.e., 1.4% of total wolf detections: see main text). Eventually, we (*i*) tested whether temporal activity of each species differed between “high human” and “low human” sites through the Watson’s two-Sample tests of homogeneity, (*ii*) calculated intra-specific overlap of temporal activity patterns of each species between “high human” and “low human” sites through the non-parametric overlap coefficient, and (*iii*) calculated interspecific coefficients of temporal overlap separately for “high human sites” and “low human sites”. These analyses were conducted at the seasonal scale (spring: April-June; summer: July-September; autumn: October-December; winter: January-March). As to coefficients of intra- and interspecific temporal overlap, we computed Δ_4_ coefficients when sample size was greater than 75 detections for both components of the pair; we computed Δ_1_ coefficients when sample size was lower than 75 detections for at least one component of the pair (Meredith & Ridout 2021). The number of detections divided into “high human” and “low human” sites is shown in Table S1.

There was no support for differences in temporal activity patterns of badger and *Martes* spp. between “high human” and “low human” sites (Table S2). There was support for differences in temporal activity patterns of wolf and fox between “high human” and “low human” sites in summer, autumn, and winter, but not in spring (Table S2). These differences were generally given by a slightly higher diurnal activity in sites with “low human” sites than in “high human” sites, especially for the wolf and the red fox (Figure S8). Nevertheless, for all the carnivore species, temporal activity patterns showed a great overlap between sites with high or low human activity, i.e., Δ_4_ coefficients ranged between 0.80 and 0.95, overall (Figure S8).

Temporal overlap with the wolf was generally consistent between “high human” and “low human” sites for all mesocarnivores. In fact, Δ_4_ coefficients were 0.84-0.90 (fox), 0.69-0.76 (badger), and 0.73-0.90 (*Martes* spp.) in sites with high human activity, and 0.88-0.93 (fox), 0.74-0.82 (badger), and 0.79-0.91 (*Martes* spp.) in sites with low human activity (Figure S9), with differences between sites ranging between 0-9%.

**Table S1** Number of detections of carnivores in sites with “high human activity” and “low human activity” throughout the study period.

| **Species** | **Spring** | | **Summer** | | **Autumn** | | **Winter** | |
| --- | --- | --- | --- | --- | --- | --- | --- | --- |
|  | **High** | **Low** | **High** | **Low** | **High** | **Low** | **High** | **Low** |
| **Wolf** | 119 | 289 | 97 | 318 | 260 | 595 | 155 | 547 |
| **Red fox** | 150 | 618 | 349 | 1593 | 437 | 1757 | 504 | 2356 |
| **Badger** | 10 | 84 | 20 | 199 | 22 | 278 | 59 | 555 |
| ***Martes* spp.** | 14 | 141 | 50 | 162 | 44 | 131 | 34 | 223 |

**Table S2** Results of Watson’s tests comparing temporal activity patterns of carnivores between in sites with “high human activity” and “low human activity” in each season. In bold, statistically significant differences.

| **Species** | ***U* (*p-value*)** | | | |
| --- | --- | --- | --- | --- |
|  | **Spring** | **Summer** | **Autumn** | **Winter** |
| **Wolf** | 0.097 (*p* > 0.05) | **0.363(*p* < 0.001)** | **0.475 (*p* < 0.001)** | **0.193 (*p* < 0.05)** |
| **Red fox** | 0.042 (*p* > 0.05) | **0.279 (*p* < 0.01)** | **0.441 (*p* < 0.001)** | **0.668 (*p* < 0.001)** |
| **Badger** | 0.041 (*p* > 0.05) | 0.081 (*p* > 0.05) | 0.052 (*p* > 0.05) | 0.137 (*p* > 0.05) |
| ***Martes* spp.** | 0.028 (*p* > 0.05) | 0.118 (*p* > 0.05) | 0.066 (*p* > 0.05) | 0.056 (*p* > 0.05) |

**Fig. S8** Overlap of temporal activity patterns of wolf, red fox, badger, and *Martes* spp., between sites with high human (solid line) and low human activity (dotted line), at the seasonal scale. The overlap area is denoted by the blue color. Coefficients of temporal overlap (wolf and fox: Δ_4_; badger and martens: Δ_1_) are shown; in brackets, 0.95 confidence intervals of coefficients estimated through bootstrap resampling (1000 replicates).

**Fig. S9** Overlap of temporal activity patterns of wolf, red fox, badger, and *Martes* spp., between sites with high human (up) and low human activity (down), at the seasonal scale. Coefficients of temporal overlap (wolf and fox in both sites; badger and *Martes* spp. in sites with low human activity: Δ_4_; badger and *Martes* spp. in sites with high human activity: Δ_1_) are shown; 0.95 confidence intervals of coefficients estimated through bootstrap resampling (1000 replicates) are also shown.

**References**

Meredith M, Ridout M. Overlap: estimates of coefficient of overlapping for animal activity patterns. 2021; https://cran.r-project.org/web/packages/overlap/overlap.pdf.

Mori E, Bagnato S, Serroni P, Sangiuliano A, Rotondaro F, Marchianò V, Cascini V, Poerio L, Ferretti F. Spatiotemporal mechanisms of coexistence in a European mammal community in a protected area of southern Italy. J Zool 2020;310:232–245.

Oberosler V, Groff C, Iemma A, Pedrini P, Rovero F. The influence of human disturbance on occupancy and activity patterns of mammals in the Italian Alps from systematic camera trapping. Mamm Biol 2017; 87:50–61.

Rossa M, Lovari S, Ferretti F. Spatiotemporal patterns of wolf, mesocarnivores and prey in a Mediterranean area. Behav Ecol Sociobiol 2021;75:32.
