# Supplementary material for: Interactions between carnivore species: limited spatiotemporal partitioning between apex predator and smaller carnivores in a Mediterranean protected area
Source: Front Zool. 2023 May 25;20:20. doi: 10.1186/s12983-023-00489-w (PMC10210480; doi:10.1186/s12983-023-00489-w)
Supplement: Supplementary file 5 — Additional file 5: Interspecific temporal overlapin sites with high vs. low wolf activity. [file 12983_2023_489_MOESM5_ESM.docx]

**Title:** Interactions between carnivore species: limited spatiotemporal partitioning between apex predator and smaller carnivores in a Mediterranean protected area

**Author list:** Francesco Ferretti^1,2*^, Raquel Oliveira^1^, Mariana Rossa^3^, Irene Belardi^1^, Giada Pacini^1^, Sara Mugnai^1^, Niccolò Fattorini^1^ & Lorenzo Lazzeri^1^

**Affiliations:** ^1^Research Unit of Behavioural Ecology, Ethology and Wildlife Management – Department of Life Sciences – University of Siena. Via P.A. Mattioli 4, 53100, Siena, Italy; ^2^NBFC, National Biodiversity Future Center, Palermo 90133, Italy; ^3^CESAM, Department of Biology, University of Aveiro, Campus de Santiago, 3810-193 Aveiro, Portugal

**Corresponding author:** Francesco Ferretti, Research Unit of Behavioural Ecology, Ethology and Wildlife Management – Department of Life Sciences – University of Siena. Via P.A. Mattioli 4, 53100, Siena, Italy. E-mail: [francesco.ferretti@unisi.it](about:blank).

**Additional file 5**

We evaluated whether temporal activity patterns of mesocarnivores and their overlap with the wolf differed between sites with high *vs.* low wolf activity (Mori et al. 2020; Rossa et al. 2021). For these analyses, we did not consider a location sampled in the second and third year, because the camera was deployed at a height of 2.9 m, to prevent the risk of theft because of logistical constraints. In 16 trapping months, this camera obtained only eight detections of red fox, one detection of badger and no *Martes* spp. detection, as well as 38 wolf detections (i.e., 1.4% of total wolf detections: see main text). For each yearly study period and for each location, we calculated the wolf detection rate as ratio of the number of wolf detections over the number of days with camera operating. Then, for each year we calculated the mean wolf detection rate across locations, and defined as “high wolf” sites those with wolf detection rate ≥ the mean value, and “low wolf” sites those with wolf detection rate < mean (Oberosler et al. 2017; Mori et al. 2020; Rossa et al. 2021). In this way, we defined as “high wolf” sites 5 locations in the first period, 20 locations in the second period, and 21 locations in the third period. Eventually, we (*i*) tested whether temporal activity of each species differed between “high wolf” and “low wolf” sites through the Watson’s two-Sample tests of homogeneity, (*ii*) calculated interspecific overlap of temporal activity patterns of between each mesocarnivore species and the wolf, separately for “high wolf” and “low wolf” sites, through the non-parametric overlap coefficient. These analyses were conducted at the seasonal scale (spring: April-June; summer: July-September; autumn: October-December; winter: January-March). As to coefficients of intra- and interspecific temporal overlap, we computed Δ_4_ coefficients when sample size was greater than 75 detections for both components of the pair; we computed Δ_1_ coefficients when sample size was lower than 75 detections for at least one component of the pair (Meredith & Ridout 2021).

The number of detections divided into “high wolf” and “low wolf” sites is shown in Table S3. There was support for differences in temporal activity patterns of wolf and fox between “high wolf” and “low wolf” sites, being these differences statistically significant in three out of four seasons (Table S4). There was no statistically significant difference between temporal activity of badger and *Martes* spp. between sites with “high wolf” and “low wolf” activity (Table S4).

Coefficients of temporal overlap between the wolf and the fox were always greater than 0.80, except for spring in “low wolf” sites, where it was 0.72 (Figure S10). Overall, Δ_1_ or Δ _4_ coefficients were *c.* 2-13% greater in “high wolf” than in “low wolf” sites, thus not supporting a greater temporal avoidance in the former sites than in the latter ones.

Coefficients of temporal overlap between the wolf and the badger were greater than 0.75 with the exceptions of spring and winter in “low wolf” sites, where they were 0.58 and 0.67, respectively (Figure S10). Overall, Δ_1_ or Δ _4_ coefficients were *c.* 2-41% greater in “high wolf” than in “low wolf” sites, thus not supporting a greater temporal avoidance in the former sites than in the latter ones.

Coefficients of temporal overlap between the wolf and *Martes* spp. were greater than 0.75 with the exceptions of spring (0.69-0.71, in “low wolf” and “high wolf” sites) and autumn-winter in “low wolf” sites, where they were 0.74 (Figure S10). Overall, Δ_1_ or Δ _4_ coefficients were *c.* 10% greater in “high wolf” than in “low wolf” sites in autumn, with differences of 2% in winter; conversely, overlap was 3-5% lower in the “high wolf” than in the “low wolf” sites in spring-summer (Figure S10).

**Table S3** Number of detections of carnivores in sites with “high wolf activity” and “low wolf activity” throughout the study period.

| **Species** | **Spring** | | **Summer** | | **Autumn** | | **Winter** | |
| --- | --- | --- | --- | --- | --- | --- | --- | --- |
|  | **High** | **Low** | **High** | **Low** | **High** | **Low** | **High** | **Low** |
| **Wolf** | 397 | 73 | 349 | 121 | 761 | 190 | 562 | 174 |
| **Red fox** | 379 | 418 | 813 | 1301 | 1021 | 1221 | 1607 | 1322 |
| **Badger** | 44 | 50 | 65 | 162 | 134 | 166 | 288 | 332 |
| ***Martes* spp.** | 17 | 138 | 36 | 188 | 28 | 147 | 84 | 173 |

**Table S4** Results of Watson’s tests comparing temporal activity patterns of carnivores between in sites with “high wolf activity” and “low wolf activity” in each season. In bold, significant differences.

| **Species** | ***U* (*p-value*)** | | | |
| --- | --- | --- | --- | --- |
|  | **Spring** | **Summer** | **Autumn** | **Winter** |
| **Wolf** | **0.454 (*p* < 0.001)** | 0.074 (*p* > 0.05) | **0.321 (*p* < 0.01)** | **0.376 (*p* < 0.01)** |
| **Red fox** | **0.460 (*p <* 0.001)** | **0.402 (*p* < 0.001)** | **0.505 (*p* < 0.001)** | 0.116 (*p* > 0.05) |
| **Badger** | 0.100 (*p* > 0.05) | 0.043 (*p* > 0.05) | 0.095 (*p* > 0.05) | 0.149 (*p* > 0.05) |
| ***Martes* spp.** | 0.134 (*p* > 0.05) | 0.050 (*p* > 0.05) | 0.031 (*p* > 0.05) | 0.073 (*p* > 0.05) |

**Fig. S10** Overlap of temporal activity patterns of wolf, red fox, badger, and *Martes* spp., between sites with high wolf and low wolf activity, at the seasonal scale. Coefficients of temporal overlap (Δ_4_ or Δ_1_ depending on sample size) are shown; error bars indicate 0.95 confidence intervals of coefficients estimated through bootstrap resampling (1000 replicates).

**References**

Meredith M, Ridout M. Overlap: estimates of coefficient of overlapping for animal activity patterns. 2021; https://cran.r-project.org/web/packages/overlap/overlap.pdf.

Mori E, Bagnato S, Serroni P, Sangiuliano A, Rotondaro F, Marchianò V, Cascini V, Poerio L, Ferretti F. Spatiotemporal mechanisms of coexistence in a European mammal community in a protected area of southern Italy. J Zool 2020;310:232–245.

Oberosler V, Groff C, Iemma A, Pedrini P, Rovero F. The influence of human disturbance on occupancy and activity patterns of mammals in the Italian Alps from systematic camera trapping. Mamm Biol 2017; 87:50–61.

Rossa M, Lovari S, Ferretti F. Spatiotemporal patterns of wolf, mesocarnivores and prey in a Mediterranean area. Behav Ecol Sociobiol 2021;75:32.
