# Supplementary material for: Interactions between carnivore species: limited spatiotemporal partitioning between apex predator and smaller carnivores in a Mediterranean protected area
Source: Front Zool. 2023 May 25;20:20. doi: 10.1186/s12983-023-00489-w (PMC10210480; doi:10.1186/s12983-023-00489-w)
Supplement: Supplementary file 6 — Additional file 6: Preliminary analyses on spatial relationships between the wolf and mesocarnivores. [file 12983_2023_489_MOESM6_ESM.docx]

**Title:** Interactions between carnivore species: limited spatiotemporal partitioning between apex predator and smaller carnivores in a Mediterranean protected area

**Author list:** Francesco Ferretti^1,2*^, Raquel Oliveira^1^, Mariana Rossa^3^, Irene Belardi^1^, Giada Pacini^1^, Sara Mugnai^1^, Niccolò Fattorini^1^ & Lorenzo Lazzeri^1^

**Affiliations:** ^1^Research Unit of Behavioural Ecology, Ethology and Wildlife Management – Department of Life Sciences – University of Siena. Via P.A. Mattioli 4, 53100, Siena, Italy; ^2^NBFC, National Biodiversity Future Center, Palermo 90133, Italy; ^3^CESAM, Department of Biology, University of Aveiro, Campus de Santiago, 3810-193 Aveiro, Portugal

**Corresponding author:** Francesco Ferretti, Research Unit of Behavioural Ecology, Ethology and Wildlife Management – Department of Life Sciences – University of Siena. Via P.A. Mattioli 4, 53100, Siena, Italy. E-mail: [francesco.ferretti@unisi.it](about:blank).

**Additional file 6**

*Preliminary analyses: effects of site-specific covariates on detection rates*

Effects of height at which camera traps were deployed, shrub cover, and canopy cover in a 10 m radius around the camera trapping site, on detection rates of focal mesocarnivores were estimated through generalized linear mixed models with negative binomial errors. For each mesocarnivore (red fox, badger, *Martes* spp.) we included the number of monthly detections in each site as response variable. The above mentioned variables were included as predictors. Camera trapping site and camera model were included as random effects. The log(sampling effort) was included as offset variable to standardize the number of detections for number of days with cameras actually operating. Effects are shown in Table S5. We considered as statistically supported effects of those variables for which 0.95 confidence intervals did not include ‘0’. Thus, we retained shrub cover (badger) and canopy cover (*Martes* spp.) for subsequent model selection.

**Table S5.** Effects of detection covariates on red fox detection rate estimated through Generalized Linear Mixed Models with negative binomial errors.

| **Mesocarnivore** | **Parameter** | ***B*** | **S.E.** | **Lower** | **Upper** |
| --- | --- | --- | --- | --- | --- |
| Red fox | Intercept | -0.922 | 0.212 | -1.338 | -0.506 |
|  | Height | -0.164 | 0.098 | -0.355 | 0.028 |
|  | Shrub cover | 0.019 | 0.102 | -0.181 | 0.219 |
|  | Canopy cover | -0.196 | 0.102 | -0.396 | 0.004 |
| Badger | Intercept | -3.163 | 0.241 | -3.635 | -2.691 |
|  | Height | -0.239 | 0.145 | -0.524 | 0.046 |
|  | Shrub cover | 0.352 | 0.149 | 0.061 | 0.643 |
|  | Canopy cover | -0.053 | 0.153 | -0.353 | 0.248 |
| *Martes* spp. | Intercept | -3.609 | 0.363 | -4.321 | -2.898 |
|  | Height | -0.315 | 0.177 | -0.661 | 0.032 |
|  | Shrub cover | -0.258 | 0.172 | -0.596 | 0.079 |
|  | Canopy cover | 0.353 | 0.173 | 0.015 | 0.692 |

*Preliminary analyses: effects of site-specific covariates on detection rates*

There was no support for a variation of carnivore and human detection rates with increasing sampling effort, as no correlation was found between carnivore or human detection rates and the number of working days (wolf: *r* = 0.035 *df* = 841, *p* = 0.305; red fox: *r* = 0.034, *df* = 841, *p* = 0.323; badger: *r* = 0.047, *df* = 841, *p* = 0.171; *Martes* spp.: *r* = -0.006, *df* = 841, *p* = 0.870; humans: *r* = -0.004, *df* = 841, *p* = 0.913; Figure S11).

**Fig. S11** Plots showing carnivore and human detection rate *vs.* number of days with cameras working.

*Preliminary analyses: list of predictors included in global models*

Table S6 shows the list of variables entered as predictors in global models, as well as the rational for including them.

**Table S6** List of variables entered as predictors in models evaluating factors influencing monthly detection rates of mesocarnivores.

| **Predictor** | **Reason** |
| --- | --- |
| Wolf detection rate | Testing the support for a negative/positive association between spatial patterns of mesocarnivore detection rates and those of the wolf. |
| Mesocarnivore detection rate | Testing the support for a negative/positive association between spatial patterns of mesocarnivore detection rates and those of the other mesocarnivore species. |
| Human detection rate | Testing whether spatial patterns of mesocarnivore detection rates are negatively or positively associated with those of humans. |
| Habitat | Testing whether detection rates of focal species are influenced by the habitat type in the camera trapping site. |
| Season | Testing whether detection rates of focal species are influenced by seasonality (e.g., they could be lower in spring – i.e. during the denning seasons – and higher in winter, i.e. during the mating season). |
| Study year | Testing whether detection rates of focal species are influenced by year (e.g., they could decrease from the first to the third year along with the increase of wolf numbers, if the latter negatively affected the former). |
| Shrub cover (for badger) | Testing whether badger detection rates were affected by shrub cover at the camera trapping site. |
| Canopy cover (for *Martes* spp.) | Testing whether *Martes* spp. detection rates were affected by canopy cover at the camera trapping site. |

*Preliminary analyses: collinearity among predictor variables*

There was no evidence for a correlation between predictor variables: all correlation coefficients were < |0.6|, except for number of detections of each study species and relevant detection rates, that were never included as predictors in the same models (Figure S12).

**
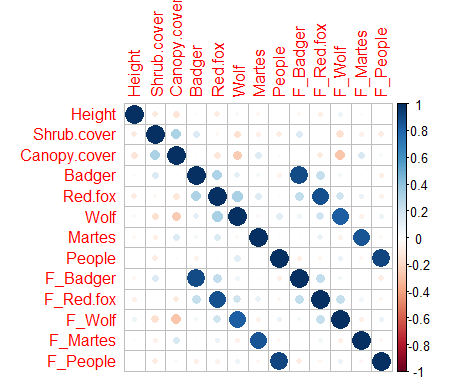
**

**Fig. S12** Correlation matrix among variables. The color bar indicates the gradient in correlation coefficients.

*Spatial variation of red fox, badger and Martes spp. detection rate: other selected models*

**Table 7.** Factors influencing spatial variation of red fox *Martes* spp. detection rates.

| **Species** | **Model** | **Variable** | ***B*** | **S.E.** | **0.95 CIs** | |
| --- | --- | --- | --- | --- | --- | --- |
|  |  |  |  |  | **-** | **+** |
| **Red fox** | **Second** | Intercept | -1.323 | 0.192 | -1.699 | -0.947 |
|  |  | Wolf | 0.092 | 0.042 | 0.009 | 0.175 |
|  |  | Badger | 0.240 | 0.047 | 0.147 | 0.333 |
|  |  | Season [Summer] | 0.450 | 0.108 | 0.238 | 0.661 |
|  |  | Season [Autumn] | 0.245 | 0.109 | 0.033 | 0.458 |
|  |  | Season [Winter] | 0.403 | 0.110 | 0.187 | 0.619 |
|  | **Third** | Intercept | -0.972 | 0.255 | -1.472 | -0.473 |
|  |  | Badger | 0.243 | 0.048 | 0.149 | 0.336 |
|  |  | Study year [Second] | -0.261 | 0.152 | -0.558 | 0.036 |
|  |  | Study year [Third] | -0.374 | 0.153 | -0.673 | -0.074 |
|  |  | Season [Summer] | 0.431 | 0.108 | 0.219 | 0.643 |
|  |  | Season [Autumn] | 0.251 | 0.108 | 0.039 | 0.464 |
|  |  | Season [Winter] | 0.393 | 0.111 | 0.176 | 0.609 |
| ***Martes* spp.** | **Second** | Intercept | -2.987 | 0.429 | -3.828 | -2.146 |
|  |  | Red fox | 0.265 | 0.068 | 0.132 | 0.397 |
|  |  | Season [Summer] | -0.143 | 0.192 | -0.519 | 0.233 |
|  |  | Season [Autumn] | -0.603 | 0.197 | -0.988 | -0.218 |
|  |  | Season [Winter] | -0.377 | 0.194 | -0.758 | 0.004 |
|  |  | Study year [Second] | -0.769 | 0.288 | -1.334 | -0.205 |
|  |  | Study year [Third] | -0.427 | 0.298 | -1.010 | 0.157 |
|  |  | Canopy cover | 0.366 | 0.167 | 0.039 | 0.694 |

**Table S7 legend:** Variables influencing spatial variation of detection rates of red fox, badger and *Martes* spp.. Spatial variation of detection rates was estimated through Generalised Linear Mixed Models with negative binomial errors. Variables included in the second selected model are shown. Estimated coefficients and their standard error as well as 0.95 confidence intervals are shown.
