# Supplementary material for: Interactions between carnivore species: limited spatiotemporal partitioning between apex predator and smaller carnivores in a Mediterranean protected area
Source: Front Zool. 2023 May 25;20:20. doi: 10.1186/s12983-023-00489-w (PMC10210480; doi:10.1186/s12983-023-00489-w)
Supplement: Supplementary file 7 — Additional file 7: Wolf-mesocarnivore spatial relationships in the same area sampled in the first study year. [file 12983_2023_489_MOESM7_ESM.docx]

**Title:** Interactions between carnivore species: limited spatiotemporal partitioning between apex predator and smaller carnivores in a Mediterranean protected area

**Author list:** Francesco Ferretti^1,2*^, Raquel Oliveira^1^, Mariana Rossa^3^, Irene Belardi^1^, Giada Pacini^1^, Sara Mugnai^1^, Niccolò Fattorini^1^ & Lorenzo Lazzeri^1^

**Affiliations:** ^1^Research Unit of Behavioural Ecology, Ethology and Wildlife Management – Department of Life Sciences – University of Siena. Via P.A. Mattioli 4, 53100, Siena, Italy; ^2^NBFC, National Biodiversity Future Center, Palermo 90133, Italy; ^3^CESAM, Department of Biology, University of Aveiro, Campus de Santiago, 3810-193 Aveiro, Portugal

**Corresponding author:** Francesco Ferretti, Research Unit of Behavioural Ecology, Ethology and Wildlife Management – Department of Life Sciences – University of Siena. Via P.A. Mattioli 4, 53100, Siena, Italy. E-mail: [francesco.ferretti@unisi.it](about:blank).

**Additional file 7**

*Models for mesocarnivore detection rates in the same area sampled in the first study year*

For red fox and *Martes* spp., models of spatial patterns were recalculated considering only camera traps located in the area sampled during the first study year (*c.* 30 km^2^, north Uccellina Mts.). Results supported an effect of the study year for stone marten/pine marten only, but not for the red fox, indicating no evidence for a decrease of fox detection rates throughout study years (Table S8).

**Table S8.** Summary of model selection. Variables influencing spatial patterns of locomotory activity of red fox and *Martes* spp., estimated through Generalized Linear Mixed Models. Predictors included in selected models are shown. All models included the random effects of camera trapping location and camera model; the log(number of sampling days) was included as offset variable. For the red fox and *Martes* spp., two and three models were selected, respectively, and were shown in bold; the third and fourth ranked models were also shown for comparison purposes.

| **Response variable** | **Model** | **Variables** | ***K*** | **logLik** | **AICc** | **ΔAICc** | **Weight** |
| --- | --- | --- | --- | --- | --- | --- | --- |
| Red fox detection rate | **Best** | **Wolf + Badger + Season** | **9** | **-1485.465** | **2989.3** | **0.00** | **0.677** |
|  | **Second** | **Badger + Season** | **8** | **-1487.264** | **2990.8** | **1.52** | **0.317** |
|  | Third | Wolf + Badger | 6 | -1496.099 | 3004.4 | 15.07 | 0.003 |
|  | Fourth | Badger | 5 | -1498.273 | 3006.7 | 17.37 | 0.003 |
| *Martes* spp. detection rate | **Best** | **Red fox + Humans + Season + Study year** | **11** | **-556.544** | **1135.6** | **0.00** | **0.355** |
|  | **Second** | **Red fox + Humans + Season** | **9** | **-558.750** | **1135.9** | **0.24** | **0.316** |
|  | **Third** | **Red fox + Season** | **8** | **-560.166** | **1136.6** | **1.00** | **0.211** |
|  | Fifth | Red fox + Humans | 6 | -562.848 | 1137.9 | 2.24 | 0.118 |
